# Daily home fortification with iron as ferrous fumarate versus NaFeEDTA: a randomised, placebo-controlled, non-inferiority trial in Kenyan children

**DOI:** 10.1186/s12916-017-0839-z

**Published:** 2017-04-28

**Authors:** Emily M. Teshome, Pauline E. A. Andang’o, Victor Osoti, Sofie R. Terwel, Walter Otieno, Ayşe Y. Demir, Andrew M. Prentice, Hans Verhoef

**Affiliations:** 1 MRCG Keneba at MRC Unit, Banjul, The Gambia; 2 MRC International Nutrition Group, Faculty of Epidemiology and Population Heath, London School of Hygiene and Tropical Medicine, Keppel Street, London, WC1E 7HT England UK; 3 Maseno University, School of Public Health and Community Development, Maseno, Kenya; 4 International Centre of Insect Physiology and Ecology, Nairobi, Kenya; 5 Division of Human Nutrition and Cell Biology and Immunology Group, Wageningen University, Wageningen, The Netherlands; 6 Maseno University, School of Medicine, Maseno, Kenya; 7 Meander Medical Centre, Laboratory for Clinical Chemistry and Haematology, Amersfoort, The Netherlands

**Keywords:** Anaemia, Child, Pre-school, Ferric sodium EDTA, Home fortification, Iron, Non-inferiority, Meta-analysis

## Abstract

**Background:**

We aimed to show the non-inferiority of home fortification with a daily dose of 3 mg iron in the form of iron as ferric sodium ethylenediaminetetraacetate (NaFeEDTA) compared with 12.5 mg iron as encapsulated ferrous fumarate in Kenyan children aged 12–36 months. In addition, we updated a recent meta-analysis to assess the efficacy of home fortification with iron-containing powders, with a view to examining diversity in trial results.

**Methods:**

We gave chemoprevention by dihydroartemisinin-piperaquine, albendazole and praziquantel to 338 afebrile children with haemoglobin concentration ≥70 g/L. We randomly allocated them to daily home fortification for 30 days with either placebo, 3 mg iron as NaFeEDTA or 12.5 mg iron as encapsulated ferrous fumarate. We assessed haemoglobin concentration (primary outcome), plasma iron markers, plasma inflammation markers and *Plasmodium* infection in samples collected at baseline and after 30 days of intervention. We conducted a meta-analysis of randomised controlled trials in pre-school children to assess the effect of home fortification with iron-containing powders on anaemia and haemoglobin concentration at end of intervention.

**Results:**

A total of 315 children completed the 30-day intervention period. At baseline, 66.9% of children had inflammation (plasma C-reactive protein concentration >5 mg/L or plasma *α*_1_-acid glycoprotein concentration >1.0 g/L); in those without inflammation, 42.5% were iron deficient. There was no evidence, either in per protocol analysis or intention-to-treat analysis, that home fortification with either of the iron interventions improved haemoglobin concentration, plasma ferritin concentration, plasma transferrin receptor concentration or erythrocyte zinc protoporphyrin-haem ratio. We also found no evidence of effect modification by iron status, anaemia status and inflammation status at baseline. In the meta-analysis, the effect on haemoglobin concentration was highly heterogeneous between trials (*I* ^2^: 84.1%; *p* value for test of heterogeneity: <0.0001).

**Conclusions:**

In this population, home fortification with either 3 mg iron as NaFeEDTA or 12.5 mg iron as encapsulated ferrous fumarate was insufficiently efficacious to assess non-inferiority of 3 mg iron as NaFeEDTA compared to 12.5 mg iron as encapsulated ferrous fumarate. Our finding of heterogeneity between trial results should stimulate subgroup analysis or meta-regression to identify population-specific factors that determine efficacy.

**Trial Registration:**

The trial was registered with ClinicalTrials.gov (NCT02073149) on 25 February 2014.

**Electronic supplementary material:**

The online version of this article (doi:10.1186/s12916-017-0839-z) contains supplementary material, which is available to authorized users.

## Background

In 2011, the World Health Organisation (WHO) recommended daily home fortification with iron (12.5 mg as a ferrous salt) in populations where the prevalence of anaemia in children younger than 5 years of age is ≥20% [1], which covers most developing countries [2]. This recommendation was based on a meta-analysis of randomised controlled trials showing moderate quality evidence for an effect on anaemia and haemoglobin concentration [3].

The WHO-recommended dose of 12.5 mg iron was established to meet almost 90% of the estimated total iron requirement of children aged 6–18 months [4]. Several trials have shown, however, that supplementation or food fortification with iron at this dose can increase rates of hospital admissions [5] as well as diarrheal and respiratory diseases [6]. In addition, it can produce a potentially more pathogenic gut microbiota profile that is associated with gut inflammation [7, 8]. Ingestion of ferrous salts also frequently causes mild gastrointestinal adverse effects (e.g. constipation, nausea, vomiting and epigastric discomfort) that may reduce adherence to treatment [9]. The frequency and severity of such effects depend on dose and dosage schedule [10, 11], may be due to oxidative stress [12] and appear to be reduced when iron is taken with food.

By comparison, ingestion of a low dose of highly bioavailable iron (3 mg iron as ferric sodium ethylenediaminetetraacetate (NaFeEDTA)) may result in similar or even higher quantities of absorbed iron [13] and may be non-inferior in its effect on iron status. It may have the advantage that reduced amounts of ingested iron may reduce proliferation of pathogenic gut bacteria, produce less oxidative stress and increase tolerability and adherence [14]. In addition, iron as NaFeEDTA has been reported to cause less oxidative stress than an equimolar dose of iron as ferrous sulphate [15]. No study has so far compared the efficacy of daily home iron fortification with 12.5 mg iron as encapsulated ferrous fumarate versus 3 mg iron as NaFeEDTA.

In malaria-endemic areas, WHO has recommended that iron interventions should be implemented in conjunction with measures to control malaria [1], because there is substantial evidence that iron interventions can increase malaria rates in young children [16].

We aimed to show non-inferiority of home fortification with 3 mg iron as NaFeEDTA compared with 12.5 mg iron as encapsulated ferrous fumarate in children aged 12–36 months under cover of chemoprevention against malaria. We conducted a pre-specified analysis to explore to what extent efficacy depended on baseline iron markers (haemoglobin concentration, plasma concentrations of ferritin and soluble transferrin receptor measured), because iron absorption is known to depend on iron status. Lastly, we used results from our present study and other recent reports to update the meta-analysis by Salam et al. [5], with a view to examining diversity in study results.

## Methods

Details of study methods are described in the statistical analysis plan (Additional file 1). During study implementation, there were no major amendments to the protocol.

### Study setting and subjects

Fieldwork was conducted between January 2014 and December 2014 in Kisumu West District, Kenya. Malaria is highly endemic in the area [17, 18], with virtually all infections due to *Plasmodium falciparum*. In children aged 1–4 years, the prevalence of infections due to *P. falciparum* has been reported to range between 39% and 63% [19]. Previous trials in the area have shown that iron supplementation resulted in increased haemoglobin concentrations among pre-school children, suggesting that iron deficiency is common [20–22].

### Study design

This was a randomised, double-blind, non-inferiority trial with three arms: 3 mg iron as NaFeEDTA (experimental treatment); 12.5 mg iron as encapsulated ferrous fumarate (active control treatment); and placebo. We included the placebo arm to demonstrate superiority of the investigational drug over placebo (proof of efficacy) [23]. The study was conceived as an explanatory trial to evaluate efficacy with maximal compliance.

### Data collection timelines and field procedures

Community health workers invited parents of children aged 12–36 months old for screening at the research clinic. During screening, research assistants measured height and length within 0.1 cm using wooden measuring boards (UNICEF, Copenhagen, Denmark) and weight to the nearest 100 g using a Salter scale (UNICEF, Copenhagen, Denmark). They also administered a standardised form to collect vital data and household characteristics data. Medical staff conducted a medical examination and collected venous blood (4 mL) in tubes containing Li-heparin for subsequent determination of iron biomarkers and *Plasmodium* infection. Children who attained the eligibility criteria were given pre-medications 3 days before randomisation to treatment allocation.

### Pre-medications

Medical staff administered drugs to prevent malaria and to control anaemia due to helminth infections in the subsequent intervention period. These pre-medications comprised: (1) dihydroartemisinin-piperaquine (Sigma-Tau, Rome, Italy; tablets of 40 mg of dihydroartemisinin and 320 mg of piperaquine), for 3 days at a daily target dose of 4 mg/kg body weight [24]; (2) albendazole (Indoco Remedies, Mumbai, India), for 3 days at a daily target dose of 200 mg or 400 mg for children aged 12–24 months and >24 to 36 months, respectively; (3) praziquantel (Cosmos, Nairobi, Kenya; 600-mg tablets), as a single dose at a target dose of 40 mg/kg body weight [25]. Piperaquine is eliminated slowly (mean elimination half-life: 23 days in children in Burkina Faso) [26], resulting in a protective efficacy against malaria for at least 1 month [27, 28]. Medical staff observed that the child swallowed the first dose of the pre-medication drugs at the research clinic. Parents were instructed to administer the remaining two doses of dihydroartemisinin-piperaquine and albendazole at home on the subsequent 2 days.

### Eligibility

Children were eligible for enrolment in the study after attaining the following eligibility criteria: aged 12–36 months; the child was expected to remain resident in the study area for the duration of the intervention and follow-up; no known or reported allergy to pre-medication drugs; not severely malnourished (weight-for-height z-score < –3 SD); absence of fever (axillary temperature <37.5 °C); absence of reported or suspected systemic disorders (e.g. HIV infection, tuberculosis, sickle cell disease); haemoglobin concentration ≥70 g/L; and at least one parent signed an informed consent form. Of the 433 children screened between April and July 2014, 338 children were randomised for intervention. In our sample size calculations [29–31], we specified a non-inferiority margin for haemoglobin concentration of 4.7 g/L, which we expected to preserve 50% of the reported and anticipated minimum effect of 12.5 mg ferrous fumarate (9.3 g/L, [32]; Additional file 1).

### Randomisation

Three days after the screening visit, children who met the eligibility criteria were randomised at the research clinic. We used a stratified block design to achieve group balance in size and baseline haemoglobin concentration. A person not involved in the fieldwork assigned the three treatment groups to a sequence of random permuted blocks of sizes 6 or 9 nested within two strata defined by baseline haemoglobin concentration class (<100 g/L and ≥100 g/L), using tables with random numbers and random permuted blocks. Following this scheme, two other persons not involved in the fieldwork produced a set of labels with a child’s identification number that included a letter for stratum (A or B) and a consecutive allocation number as indicated by the randomisation scheme. At the randomisation visit, the trial coordinator assigned children successively to the next available allocation number randomised for treatment and according to appropriate stratum.

### Composition of fortificants

We used three types of micronutrient powders that contained vitamin A and zinc contents as per WHO recommendations [1], 11 other micronutrients (Table 1) at doses as recommended by the Home Fortification Technical Advisory Group [33] and, in addition, either 3 mg iron as NaFeEDTA, or 12.5 mg iron as encapsulated ferrous fumarate, or no iron (placebo). The micronutrient powders were packed in 1-g plain white foil single-serve sachets that were identical in appearance and that did not result in apparent differences in taste, texture or colour of *uji* (porridge made of maize flour). We excluded folic acid because of concerns that it may cause failure of antifolate drugs and because of the absence of evidence that folate deficiency anaemia is a public health problem among children in developing countries [34].

**Table 1:** Composition of home fortificants

Micronutrient	Content
Vitamin A	300 μg RE
Vitamin D	5 μg
Vitamin E	5 mg
Vitamin C	30 mg
Thiamin (vitamin B_1_)	0.5 mg
Riboflavin (vitamin B_2_)	0.5 mg
Niacin (vitamin B_3_)	6 mg
Vitamin B_6_ (pyridoxine)	0.5 mg
Vitamin B_12_ (cobalamin)	0.9 μg
Iron	
EITHER iron as encapsulated ferrous fumarate	12.5 mg
OR iron as NaFeEDTA	3 mg
OR no iron (placebo)	0 mg
Zinc	5 mg
Copper	0.56 mg
Selenium	17 μg
Iodine	90 μg

*RE* retinol equivalents

### Blinding

Researchers, outcome assessors and parents remained blinded to the type of treatment allocated to each child until the 30-day intervention period had been completed. At this point they were partially unblinded to know if the child was in the placebo or iron group. Investigators were fully unblinded after the fieldwork was complete and the statistical analysis plan written.

### Adherence monitoring

Adherence to intervention was monitored using an electronic monitoring and time-recording device (Medication Events Monitoring Systems, MEMS, 6 TrackCap 45 mm without LCD display; WestRock, Sion, Switzerland; http://www.medamigo.com/) that is considered to be the reference standard and superior to medication counts and self-reported adherence methods [35, 36]. The battery-operated device consists of a cap with a built-in microprocessor that fits the bottle with the micronutrient sachets. It records and internally stores dates and times of all openings. Parents were taught how to use the bottle with the electronic device without knowing that the device was monitoring the openings of the bottle and instructed to return the electronic device with any remaining sachets at the end of 30 days of intervention.

### Intervention period

On the first day of intervention, research assistants gave parents a supply of 30 sachets in a plastic bottle with a MEMS cap and instructed them to add the contents of a single-serve sachet to the child’s semi-solid, ready-prepared foods every day for a period of 30 days. The main staple food consumed by pre-school children was *uji* made from locally milled flour from either maize or sorghum grains. The grains are not de-germed and sifted and so have high contents of phytic acid and phenolic compounds [37, 38]. At the research clinic, the assistants showed parents how to mix the contents of the first sachet with *uji*. This first dose was consumed at the research facility, and trained research assistants closely observed that each child consumed all the *uji*. Parents were given a mosquito net and instructed to immediately inform research assistants whenever a child fell sick. Research assistants conducted weekly pre-announced home visits to check if parents were adhering to instructions given at randomisation. Sick children found in the homes were referred to the research clinic. Children with fever (≥37 °C) and who tested positive for *Plasmodium* infection by microscopic examination of blood smears were treated immediately with artemisinin-lumefantrine; during treatment, these children were temporarily discontinued from the intervention treatment until medication was completed and subsequent microscopic examination of blood smears conducted after 7 days showed negative for malaria parasites.

Parents who withdrew children from the intervention were asked for reasons and permission to keep and analyse data and samples already collected. After 30 days of intervention, the phlebotomist collected venous blood (4 mL) and processed samples as stated above (Data collection time lines and field procedures). Medical staff examined every child, and fieldworkers collected anthropometric data, plastic bottles with the MEMS cap and empty sachets and administered a questionnaire to parents to collect additional information on possible factors affecting adherence. Once all data and samples were collected, the trial coordinator opened the sealed brown envelope to find out the child’s intervention group (either iron or placebo).

### Post-intervention period

For ethical reasons, children in the placebo group were given a 3-day course of dihydroartemisinin-piperaquine and a subsequent 30-day course of home fortification with 12.5 mg iron as encapsulated ferrous fumarate. Children in the iron group were retained without fortification powders to monitor the population decline in haemoglobin concentration over time in a 100-day follow-up period. Parents were requested to take each child home and bring them back to the research clinic on a date generated by a pre-programmed Microsoft Excel software that randomly selected a date of their return visit within a 100-day period. On the return visit, a capillary blood sample was collected by finger puncture to measure haemoglobin concentrations in duplicate from a single drop and to store DNA on collection cards for subsequent assessment by PCR assay of *Plasmodium* parasites. Immediately, these children were withdrawn from further study and received appropriate medication if sick, a therapeutic course of dihydroartemisinin-piperaquine and a supply of sachets for daily home fortification with 12.5 mg iron as encapsulated ferrous fumarate for another 30 days.

### Laboratory analysis

We determined haemoglobin concentration (HemoCue 301, Ängelholm, Sweden) and zinc protoporphyrin (ZPP)-haem molar ratio (AVIV, model 206D, Lakewood NJ, USA) in whole blood and in erythrocytes as a marker of iron-deficient erythropoiesis, each in triplicate. We assayed *Plasmodium* antigenaemia by histidine-rich protein 2 (HRP2) and lactate dehydrogenase (LDH) tests and transferred aliquots of whole blood (125 μL) on DNA collection cards (FTA Mini Card, catalogue WB120055, GE Healthcare, Little Chalfont, UK) for storage at ambient temperature and subsequent detection by PCR of *Plasmodium* infection; we also prepared thick and thin blood smears to allow for detection and counting of *Plasmodium* parasites. Iron markers (plasma concentrations of ferritin, soluble transferrin receptor and transferrin), inflammation markers (plasma concentrations of C-reactive protein (CRP) and *α*_1_-acid glycoprotein), albumin and vitamin B12 were measured at Meander Medical Centre, Amersfoort, The Netherlands, on an Abbott Architect C16000 and i2000 SR analyser as per manufacturer’s instructions.

### Statistical analysis

Details of the statistical analysis are presented in a supplementary paper (Additional file 1). Data were double entered, checked for completeness and verified for possible entry errors using Microsoft Excel. Anthropometric indices and electronic adherence data were analysed using WHO Anthro software v.3.2.2 (World Health Organisation, Geneva, Switzerland) and PowerView v.3.5.2 (AARDEX Group Ltd, Sion, Switzerland), respectively. The final statistical analysis was conducted using SPSS 21 (IBM, Armonk, NY, USA) and CIA 2.2.0 (https://eprints.soton.ac.uk/393017/). To assess EDTA intake at home fortification levels of 3 mg iron as NaFeEDTA, we calculated the intake of iron per kilogram of body weight for all children in the trial, both at baseline and at 30 days after intervention. The corresponding intake of NaFeEDTA (NaFeC_10_H_12_N_2_O_8_) was calculated as intake iron × (molecular weight_EDTA_/molecular weight_Iron_) = intake iron × (288.21/55.88). From these results, we assessed the prevalence of EDTA intake exceeding the upper level of the acceptable daily intake (ADI) (i.e. the amount of a food additive, expressed on a body weight basis, that can be ingested daily over a lifetime without appreciable health risk [39]) to be 1.9 mg/kg body weight [40].

The definitions we used were anaemia: haemoglobin concentration <110 g/L [41]; iron deficiency: plasma ferritin concentration <12 μg/L [42]; *Plasmodium* infection: presence of parasites and gametocytes of any *Plasmodium* species [43]; inflammation: plasma CRP concentration >5 mg/L [44] or plasma *α*_1_-acid glycoprotein concentration >1.0 g/L [45], respectively. Group adherence was defined as the proportion of children who consumed ≥24 home fortification doses, corresponding to ≥80% of the 30 scheduled doses [46], with exclusion of children who were lost to follow-up because they moved out of the study area.

Variation of outcomes with a log-normal distribution was expressed as geometric standard deviation (GSD), i.e. a multiplicative factor such that division or multiplication of the geometric mean by this ratio indicates a variation that is equivalent to subtraction or addition of one standard deviation on a log-transformed scale [47].

Plasma ferritin concentration can be elevated by inflammation independently of iron status. Thus, when estimating the prevalence of iron deficiency, we accounted for inflammation by two methods. First, we restricted the analysis to children without inflammation. This method has the disadvantages that it leads to a reduced statistical precision (because of the reduced sample size) and it may produce biased results (if the true prevalence of iron deficiency differs between those with and without inflammation). Second, we used linear regression to adjust plasma ferritin concentrations for plasma concentrations of CRP and *α*_1_-acid glycoprotein concentration. We used the following formula for adjustment: log_e_(ferritin_adjusted_) = log_e_(ferritin_unadjusted_) – *β*_1_[log_e_(CRP_observed_) – log_e_(CRP_reference_)] – *β*_2_ [log_e_(AGP_observed_) – log_e_(AGP_reference_)]; the results were then exponentiated to express ferritin concentrations in their natural units. The formula given here is similar to what was recently proposed [48]; however, we used log-transformed values because of the linear relationship that has been observed between log-ferritin and log-CRP, as well as between log-ferritin and log-*α*_1_-acid glycoprotein (Suchdev, personal communication, 2016; and also the present study (not shown)). We used 15 mg/L and 2.59 g/L as reference values for CRP and *α*_1_-acid glycoprotein, respectively, because these values were reported as the upper limits of the 95% reference range in healthy French children aged 3–5 years [49]. We implicitly assumed that higher upper values for reference ranges, as can be observed in their peers in developing countries, are due to infections and other inflammation-inducing disorders.

The objective of non-inferiority was determined by comparing end points obtained by both intention-to-treat and per protocol analyses. We visually inspected histograms to assess whether outcome variables were normally distributed within intervention groups. Outcome variables with a log-normal distribution were log-transformed; exponentiation of group differences in log-transformed outcomes resulted in associations being expressed as relative differences. Outcomes that were not normally distributed, even after log-transformation, were compared using non-parametric tests. We estimated effects; *p* values, where reported, are two-sided.

For the primary analysis, we estimated the difference in haemoglobin concentrations at the end of the 30-day fortification period between groups of children allocated to different iron formulations. Analysis was done using analysis of variance (ANOVA) and multiple linear regression analysis. As pre-planned, we accepted non-inferiority only when all of the following conditions were met: (1) home fortification with 3 mg iron as NaFeEDTA was superior to placebo (proof of efficacy); (2) home fortification with 12.5 mg iron as encapsulated ferrous fumarate was superior to placebo (proof of assay sensitivity); and (3) the lower limit of the 95% confidence interval (CI) around the difference in haemoglobin concentration between the groups who received home fortification with the two different iron formulations excluded the non-inferiority margin of 4.7 g/L, in both intention-to-treat and per protocol analyses (proof of efficacy).

For the secondary analysis, we used stratified analysis and multiple linear regression models to control for group imbalances of baseline factors that were strongly or moderately associated with primary outcome and were likely to influence the effect estimates. These baseline factors were plasma ferritin concentration, plasma soluble transferrin receptor concentration and age.

#### Subgroup analysis

Because iron absorption is known to depend on iron status, we considered iron markers at baseline (anaemia, iron status) as potential modifiers for intervention effects on haemoglobin concentration and plasma ferritin concentration at the end of the 30-day intervention period. Stratified analysis was used to measure effect sizes within subgroups; evidence for group differences in intervention effects was formally investigated in multiple linear regression models that included intervention and each baseline factor as main terms, as well as their product term. We conducted all three possible paired group comparisons (12.5 mg iron as ferrous fumarate versus 3 mg iron as NaFeEDTA; 12.5 mg iron as ferrous fumarate versus placebo; 3 mg iron as NaFeEDTA versus placebo). In these analyses, we adjusted for plasma concentrations of ferritin and transferrin receptor at baseline (both as continuous variables); in the analysis of plasma ferritin concentration at 30 days after start of intervention, these baseline variables were log-transformed because this gave a better model fit than the untransformed variables.

#### Meta-analysis

We conducted a meta-analysis of randomised controlled trials in pre-school children to assess the effect of home fortification with iron-containing powders on anaemia and haemoglobin concentration at end of intervention. The methods are described in Additional file 2.

## Results

### Flow of participants

**Fig. 1:**
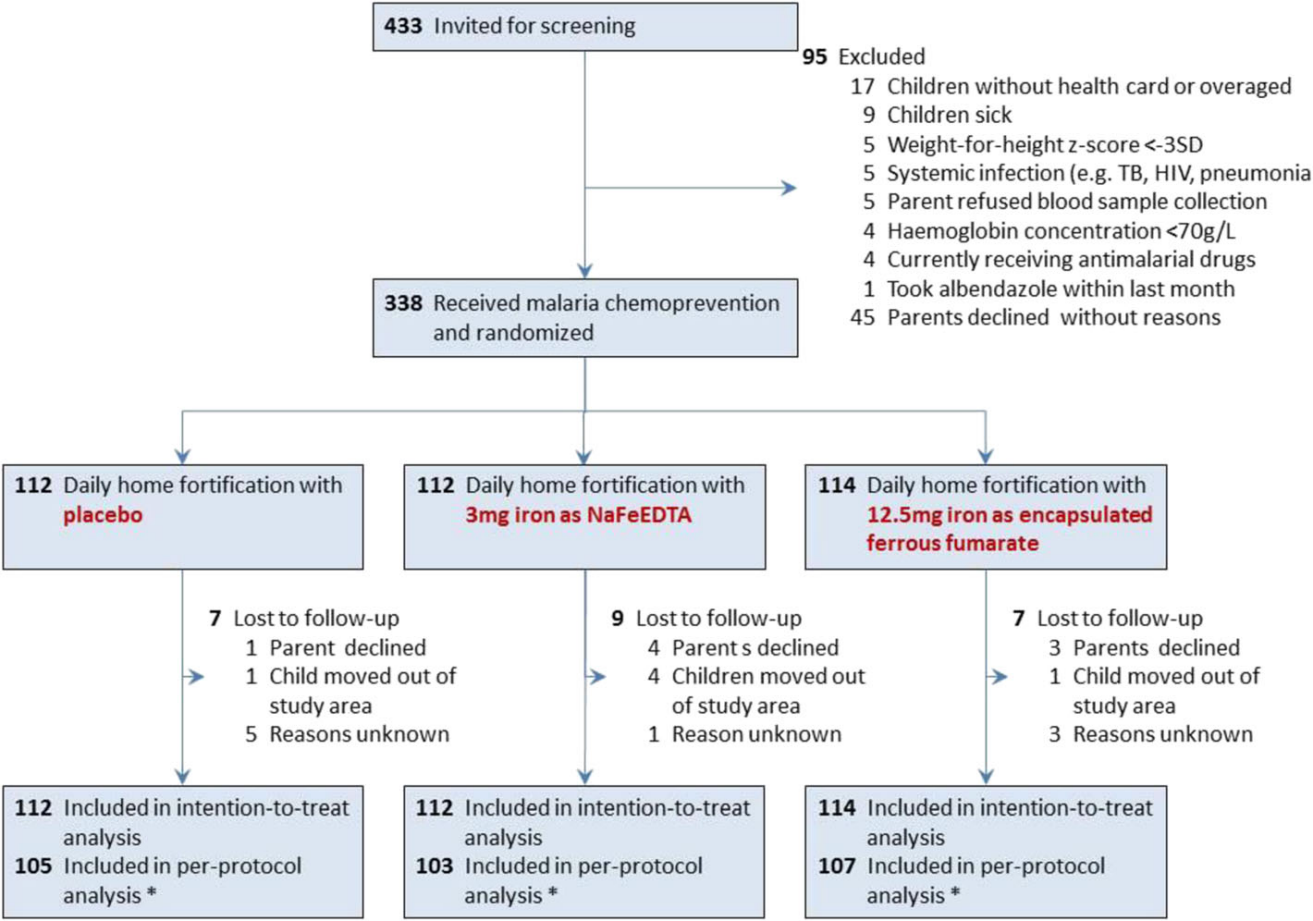
Participant flow through the trial. *Sample sizes below number indicated are due to missing values, which varied by outcome. In the intention-to-treat analysis, missing values were replaced by multiple imputation

Of 433 children who were invited for screening, 366 children met the criteria for pre-medication administration and 338 children were randomised. Of these, 315 (93.5%) completed 30 days of the intervention period and were included in the per protocol analysis (Fig. 1). Reasons for loss to follow-up were refusal by parents (*n* = 8), moving out of the study area (*n* = 6) and unknown (*n* = 9).

A total of 80 children became sick during the 30-day intervention period. We found no evidence that the proportion of sick children varied between intervention groups (*p* = 0.13). The most common infections were uncomplicated malaria with upper respiratory tract infections (24), severe malaria (14), upper respiratory tract infections (11) and uncomplicated malaria (8). Overall, 200 (60.6%) of the children consumed more than 80% (>24) of 30 scheduled sachets, and there was no evidence that this percentage differed between intervention groups (*p* = 0.99).

### Baseline characteristics

The intervention groups were similar with respect to baseline characteristics (Table 2). Overall, the mean age was 23.6 months; 62.1% of children had anaemia. Inflammation as indicated by elevated concentrations of CRP or *α*_1_-acid glycoprotein occurred in 66.9% (226/338) of children. The prevalence of iron deficiency was 17.1% (57/333) or 42.5% (57/134), depending on whether or not children with inflammation were excluded from analysis. With ferritin concentrations adjusted for inflammation by linear regression, the prevalence of iron deficiency was 53.2% (177/333). Of 366 blood samples assessed for the presence of *P. falciparum* by microscope or rapid dipstick tests (HRP2 or *P. falciparum*-specific pLDH), 40.7% and 36.4% of children were positive, respectively, with a median (25^th^, 75^th^ percentiles) density of asexual parasites of 1410 (207, 682) μ/L. The prevalence of being stunted, wasted and underweight was 30.1%, 3.3% and 13.9%, respectively.

**Table 2:** Baseline characteristics, by intervention group

Characteristic	Placebo	Iron, 3 mg as NaFeEDTA	Iron, 12.5 mg as ferrous fumarate
Number (*n*)	112	112	114
General characteristics			
Sex, male	69 (61.6%)	61 (54.5%)	56 (49.1%)
Age, months	22.8 (6.8)	23.2 (6.2)	24.9 (6.4)
Age class			
12–23 months	61 (54.5%)	57 (50.9%)	44 (38.6%)
24–36 months	51 (45.5%)	55 (49.1%)	70 (61.4%)
Nutritional markers			
Haemoglobin concentration, g/L	104.4 (13.2)	105.9 (13.3)	104.7 (13.3)
Anaemia			
Moderate (haemoglobin concentration 70–99.99 g/L)	33 (29.5%)	34 (30.4%)	36 (31.6%)
Mild (haemoglobin concentration 100–109.99 g/L)	39 (34.8%)	31 (27.7%)	37 (32.5%)
No anaemia (haemoglobin concentration ≥110 g/L)	40 (35.7%)	47 (42.0%)	41 (36.0%)
ZPP:haem ratio, μmol:mol^a^			
In whole blood	170 (119; 305)	172 (102; 260)	196 (137; 283)
In erythrocytes	130 (84; 301)	141 (76; 223)	160 (101; 246)
Plasma ferritin concentration, μg/L^a,b^	37.7 (17.3; 74.0)	31.4 (16.0; 56.6)	36.9 (17.6; 68.8)
Iron status^b^			
Deficient (plasma ferritin concentration <12 μg/L)	17 (15.2%)	20 (18.3%)	20 (17.9%)
Replete (plasma ferritin concentration ≥12 μg/L in the absence of inflammation)	26 (23.2%)	32 (29.4%)	19 (17.0%)
Uncertain (plasma ferritin concentration ≥12 μg/L in the presence of inflammation)	69 (61.6%)	57 (52.3%)	73 (65.2%)
Iron deficiency, based on adjusted ferritin concentrations^b,c^	52.7% (59/112)	57.8% (63/109)	49.1% (55/112)
Plasma soluble transferrin receptor concentration, mg/L^a^	2.41 (1.75; 3.46)	2.39 (1.82; 3.16)	2.60 (1.93; 3.41)
Plasma albumin concentration, g/L	34.6 (3.9)	35.0 (3.5)	34.7 (4.1)
Vitamin B_12_ concentration, pmol/L	391 (291; 557)	409 (311; 569)	401 (315; 554)
Infection and inflammation markers			
Plasma C-reactive protein concentration, mg/L^a^	2.5 (0.7; 7.6)	2.5 (0.6; 7.8)	4.5 (1.3; 11.0)
Plasma *α*_1_-acid glycoprotein concentration, g/L^a^	1.20 (0.90; 1.57)	1.08 (0.81; 1.47)	1.17 (0.97; 1.63)
Inflammation			
Plasma C-reactive protein concentration >5 mg/L	40 (35.7%)	35 (31.3%)	53 (46.5%)
Plasma *α*_1_-acid glycoprotein concentration >1 g/L	71 (63.4%)	63 (56.3%)	81 (71.1%)
Plasma C-reactive protein concentration >5 mg/L or plasma *α*_1_-acid glycoprotein concentration >1 g/L	77 (68.8%)	65 (58%)	84 (73.7%)
*Plasmodium* antigenaemia, by rapid dipstick tests^d^			
*P. falciparum* (either HRP2 or *P. falciparum*-specific pLDH)	39 (35.1%)	40 (36.0%)	43 (38.1%)
*Plasmodium* species other than *P. falciparum* (pLDH specific for *P. malariae*, *P. ovale* or *P. vivax*)^e^	1 (0.9%)	2 (1.8%)	0
Any *Plasmodium* species	39 (35.1%)	41 (36.9%)	43 (38.1%)
Blood smear tests, by microscopy			
Asexual or sexual forms of *P. falciparum*	47 (41.9%)	49 (43.8%)	55 (48.2%)
Asexual forms of both *P. falciparum* and human *Plasmodium* spp. other than *P. falciparum* (i.e. *P. malariae*, *P. ovale* or *P. vivax*)	2 (1.8)	1 (0.8)	2 (1.8)
Asexual parasite density for *P. falciparum*, μ/L^a^	757 (172; 3972)	3340 (297; 20,023)	1048 (207; 6820)
Low (<1000 μ/L)	17 (15.2%)	9 (8.0%)	19 (16.7%)
Medium (1000–9999 μ/L)	12 (10.7%)	8 (7.1%)	15 (13.2%)
High (≥10,000 μ/L)	3 (2.7%)	7 (6.3%)	5 (4.4%)
Gametocyte density, μ/L^a^	326 (49; 732)	82 (37; 120)	1126 (57; 2679)
Anthropometric markers			
Body height, cm	80.9 (5.9)	82.1 (5.7)	82.4 (5.1)
Body weight, kg	10.6 (1.8)	10.8 (2.0)	10.9 (1.7)
Height-for-age z-score, SD	–1.42 (1.47)	–1.15 (1.43)	–1.43 (1.31)
Weight-for-height z-score, SD	–0.10 (1.05)	–0.16 (1.07)	–0.14 (0.93)
Weight-for-age z-score, SD	–0.81 (1.18)	–0.71 (1.22)	–0.86 (1.09)
Stunted (height-for-age z-score < –2 SD)	34 (30.4%)	31 (27.7%)	37 (32.5%)
Wasted (weight-for-height z-score < –2 SD)	3 (2.7%)	5 (4.5%)	2 (1.8%)
Underweight (weight-for-age z-score < –2 SD)	19 (17.0%)	12 (10.7%)	15 (13.2%)

Values indicate *n* (%), mean (SD) or ^a^median (25th and 75th percentiles). *HRP2* histidine-rich protein-2, *pLDH P. falciparum*-specific lactate dehydrogenase

^b^Due to missing values for ferritin concentrations in 5 children, *n* was 112, 109 and 112 for groups that received placebo, NaFeEDTA and ferrous fumarate, respectively; ^c^based on ferritin concentrations adjusted for inflammation by linear regression (see text)

^d^Due to missing values in 3 children, *n* was 111, 111 and 113 for groups that received placebo, NaFeEDTA and ferrous fumarate, respectively

^e^One child in placebo group and 1 child in NaFeEDTA group with antigenaemia due to *P. malariae* or *P. ovale* or *P. vivax* also had antigenaemia to *P. falciparum* (mixed infection)

### EDTA intake

**Fig. 2:**
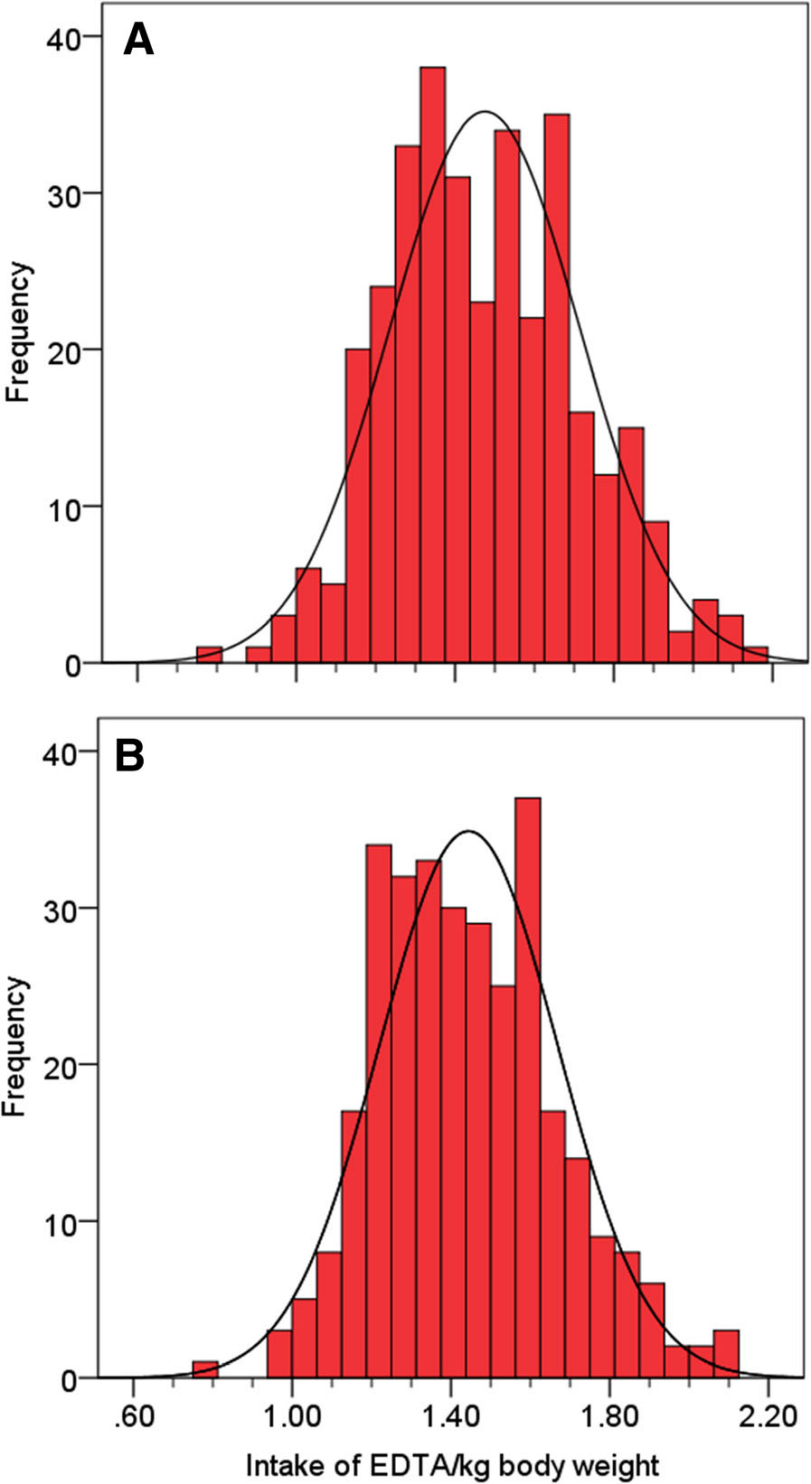
EDTA intake at daily home fortification levels of 3 mg iron as NaFeEDTA, at baseline (**a**) and at 30 days after start of intervention (**b**). The acceptable daily intake (*ADI*) for EDTA is <1.9 mg/kg body weight

Home fortification with 3 mg iron as NaFeEDTA resulted in a mean intake (SD) of 1.48 (0.24) EDTA/kg body weight and 1.44 (0.23) EDTA/kg body weight at baseline and at 30 days after the start of intervention, respectively (Fig. 2). Correspondingly, assuming that this intake follows a normal distribution, 4.0% and 2.0% of children would exceed the ADI for EDTA of 1.9 mg/kg body weight.

### Intervention effects

In the per protocol analysis, there was no evidence that home fortification for 30 days, whether using a daily dose of 3 mg iron as NaFeEDTA or 12.5 mg iron as ferrous fumarate, was efficacious in improving haemoglobin concentration, plasma ferritin concentration, plasma transferrin receptor concentration or erythrocyte ZPP-haem ratio (Table 3). Adjustment for baseline factors that were prognostic for haemoglobin concentration did not substantially change intervention effects (haemoglobin concentration difference of 1.3 g/L (–1.8 g/L to 4.3 g/L). Similarly, the intention-to-treat analysis (Additional file 3: Table S2) led to virtually identical results as obtained by per protocol analysis, and restriction of the analysis to children without inflammation led to similar effect estimates on haemoglobin concentration (0.9 g/L, –4.1 to 5.9 g/L and 2.3 g/L, –3.1 to 7.8 g/L for 3 mg iron as NaFeEDTA and 12.5 mg iron as ferrous fumarate, respectively). Given these results, we conducted no further analysis to demonstrate non-inferiority of 3 mg iron as NaFeEDTA.

**Table 3:** Effect of daily home fortification with 3 mg iron as NaFeEDTA and 12.5 mg iron as encapsulated ferrous fumarate on continuous outcomes at 30 days after start of intervention, per protocol analysis

Outcome/intervention group	No. (*n*)	Estimate^a^	Effect (95% CI) relative to placebo^b^	Effect (95% CI) relative to standard^b^
Haemoglobin concentration				
Placebo	105	106.9 g/L (13.3 g/L)	Reference	Not applicable
Iron, 3 mg as NaFeEDTA	103	110.0 g/L (12.5 g/L)	3.0 g/L (–0.2 g/L to 6.2 g/L)^c^	1.3 g/L (–1.8 g/L to 4.3 g/L)^c^
Iron, 12.5 mg as ferrous fumarate	107	108.6 g/L (12.0 g/L)	1.6 g/L (–1.6 g/L to 4.8 g/L)^c^	Reference
Plasma ferritin concentration				
Placebo	104	29.7 μg/L [3.47]	Reference	Not applicable
Iron, 3 mg as NaFeEDTA	102	33.7 μg/L [2.53]	16.2% (–14.3% to 57.7%)^d^	2.5% (–22.4% to 35.4%)^d^
Iron, 12.5 mg as ferrous fumarate	105	32.6 μg/L [3.00]	12.3% (–17.1% to 52.0%)^d^	Reference
Plasma soluble transferrin receptor concentration				
Placebo	105	2.24 mg/L [1.61]	Reference	Not applicable
Iron, 3 mg as NaFeEDTA	103	2.15 mg/L [1.47]	–4.3% (–13.5% to 5.9%)^d^	3.6% (–5.5% to 13.6%)^d^
Iron, 12.5 mg as ferrous fumarate	106	2.07 mg/L [1.38]	–7.3% (–16.2% to 2.6%)^d^	Reference
Erythrocyte ZPP-haem ratio				
Placebo	104	136 μmol/mol [2.17]	Reference	Not applicable
Iron, 3 mg as NaFeEDTA	103	127 μmol/mol [1.97]	–6.5% (–23.5% to 14.2%)^d^	-5.3% (-21.7% to 14.5%) ^d^
Iron, 12.5 mg as ferrous fumarate	106	134 μmol/mol [2.00]	–0.7% (–18.6% to 21.0%)^d^	Reference

^a^Mean (SD) or geometric mean [geometric standard deviation]

^b^Effects were adjusted for study design (blocks nested within strata of haemoglobin concentration <100 g/L and ≥100 g/L)

^c^Effects were calculated as absolute difference in means

^d^Exponentiation of group differences with log-transformed outcomes resulted in associations being expressed as relative differences

Compared to placebo, home fortification with 3 mg iron as NaFeEDTA seemed to reduce the prevalence of iron deficiency by 20.2% (Table 4); one-quarter of children who received this iron formulation nonetheless remained anaemic after 30 days. Home fortification with 12.5 mg iron as ferrous fumarate seemed to reduce the prevalence of iron deficiency by 14.1% (95% CI: –3.5 to 30.7%), but the statistical evidence for such a reduction, as judged by the 95% CI, was weak. There was no evidence that either of the iron interventions affected the prevalence of anaemia, which affected one-half of children at the end of the 30-day intervention period, or the prevalence of *Plasmodium* infection, which remained at 16.4–19.1%, depending on the method of assessment.

**Table 4:** Effect of daily home fortification with 3 mg iron as NaFeEDTA and 12.5 mg iron as encapsulated ferrous fumarate on categorical outcomes at 30 days after start of intervention, per protocol analysis

Outcome/intervention group	Prevalence	(*n*/*n*)	Effect (95% CI) relative to placebo
Anaemia			
Placebo	53.3%	(56/105)	Reference
Iron, 3 mg as NaFeEDTA	43.7%	(45/103)	–9.6% (–22.7% to 3.9%)
Iron, 12.5 mg as ferrous fumarate	51.4%	(55/107)	–1.9% (–15.1% to 11.3%)
Iron deficiency^a^			
Placebo	44.6%	(25/56)	Reference
Iron, 3 mg as NaFeEDTA	24.5%	(12/49)	–20.2% (–36.4% to –18.5%)
Iron, 12.5 mg as ferrous fumarate	30.5%	(18/59)	–14.1% (–30.7% to 3.5%)
*Plasmodium* infection, by dipstick test^b^			
Placebo	16.2%	(17/105)	Reference
Iron, 3 mg as NaFeEDTA	18.4%	(19/103)	2.3% (–8.1% to 12.6%)
Iron, 12.5 mg as ferrous fumarate	22.6%	(24/106)	6.4% (–4.3% to 17.0%)
*P. falciparum* infection, by microscopy			
Placebo	18.5%	(19/103)	Reference
Iron, 3 mg as NaFeEDTA	15.5%	(15/97)	–3.0% (–13.4% to 7.6%)
Iron, 12.5 mg as ferrous fumarate	15.2%	(15/99)	–3.3% (–13.6% to 7.2%)

^a^Analysis restricted to children without inflammation (see text)

^b^Presence of HRP, pLDH specific to *P. falciparum* or pLDH due to human *Plasmodium* species other than *P. falciparum*

### Subgroup analysis

**Fig. 3:**
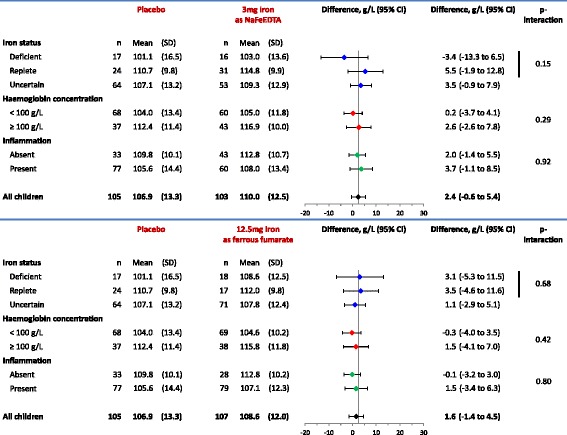
Effect of interventions on haemoglobin concentration (g/L) at 30 days after start of intervention, by subgroups of iron status and haemoglobin concentration class at baseline. Inflammation was defined to be absent when plasma C-reactive protein (*CRP*) concentration was ≤5 mg/L and plasma *α*_1_-acid glycoprotein concentration was ≤1.0 g/L, and present when either plasma CRP concentration was >5 mg/L or plasma *α*_1_-acid glycoprotein concentration was >1.0 g/L. Group means were obtained by one-way ANOVA. Intervention effects were adjusted for stratified block design as well as plasma concentrations of ferritin and soluble transferrin receptor at baseline (both continuous variables). The *p* values indicate the two-sided probability that group effects are as different as observed or more extreme when assuming that they are identical

**Fig. 4:**
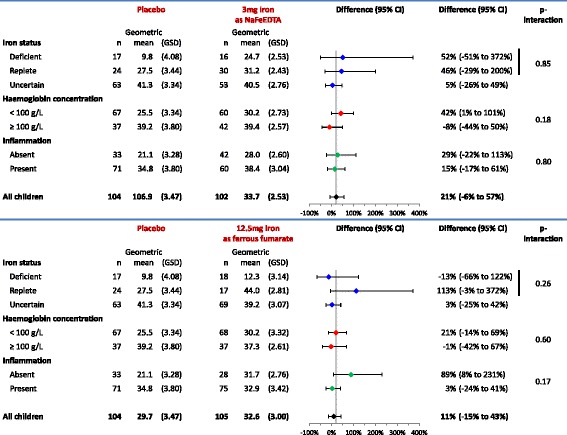
Effect of interventions on plasma ferritin concentration (μg/L) at 30 days after start of intervention, by subgroups of iron status and haemoglobin concentration class at baseline. Inflammation was defined to be absent when plasma CRP concentration was ≤5 mg/L and plasma *α*_1_-acid glycoprotein concentration was ≤1.0 g/L, and present when either plasma CRP concentration was >5 mg/L or plasma *α*_1_-acid glycoprotein concentration was >1.0 g/L. Group geometric means were obtained by exponentiation of results of one-way ANOVA. Intervention effects were adjusted for stratified block design as well as plasma concentrations of ferritin and soluble transferrin receptor at baseline (both log-transformed continuous variables). Exponentiation of group differences with log-transformed outcomes resulted in associations being expressed as relative differences. *GSD* geometric standard deviation. The *p* values indicate the two-sided probability that group effects are as different as observed or more extreme when assuming that they are identical

There was no evidence that the effect of home fortification with iron, whether administered at a daily dose of 3 mg iron as NaFeEDTA or 12.5 mg iron as encapsulated ferrous fumarate, on haemoglobin concentration or plasma ferritin concentration was modified by iron status or anaemia status at baseline (see Figs. 3 and 4). The effect of home fortification with 12.5 mg iron as ferrous fumarate on increased plasma ferritin concentration seemed larger in children without inflammation than in their peers with inflammation at baseline (89% versus 3%), but the statistical evidence of such an effect modification was weak (*p* interaction: 0.17; Fig. 4).

### Meta-analysis

The effect on haemoglobin concentration was highly heterogeneous (*I* ^2^: 8.1%; *p* value for test of heterogeneity: <0.0001; Additional file 2: Figure S4). The pooled effect on haemoglobin concentration was 3.9 g/L (95% CI: 2.2–5.50 g/L), indicating that in a random sample of a hypothetically infinite number of trials, each estimating a different true underlying effect, one may on average expect an increase in haemoglobin concentration by 3.9 g/L, with the 95% CI excluding an effect beyond 5.5 g/L.

## Discussion

We found no evidence that daily home fortification for 30 days with a daily dose of either 3 mg iron as NaFeEDTA or 12.5 mg iron as encapsulated ferrous fumarate was efficacious in improving haemoglobin concentration or iron markers (plasma ferritin concentration, plasma soluble transferrin receptor concentration or erythrocyte ZPP-haem ratio). Compared to placebo, however, home fortification with 3 mg iron as NaFeEDTA reduced the prevalence of iron deficiency by 20.2% (44.6% versus 24.5%; Table 4). Meta-analysis of trial results indicates a small, heterogeneous effect of home fortification with iron-containing powders on haemoglobin concentration.

Dichotomising a continuous outcome variable has the disadvantages that individuals close to but on opposite sides of the cut-off value are characterised as being very different rather than very similar [50], and that the prevalence difference depends on the cut-off value used for dichotomisation of the outcome [51]. Thus, in our study, a small group difference in the distribution of plasma ferritin concentration may misleadingly result in a relatively large difference in prevalence of iron deficiency. For this reason, group differences in the prevalence of iron deficiency should be interpreted with caution, and more weight should be given to group differences in plasma ferritin concentrations.

Adherence to intervention was suboptimal, with a substantial proportion of children (~40%) having consumed <80% of the scheduled sachets. We found no evidence of bias in effect estimates due to differences in baseline factors that were prognostic for haemoglobin concentration. Our failure to show efficacy for either compound was found in both the per protocol and intention-to-treat analyses, was consistent across subgroups that were defined by anaemia and iron status and precluded further assessment of the non-inferiority of 3 mg iron as NaFeEDTA compared to 12.5 mg iron as encapsulated ferrous fumarate.

Despite a course of chemoprevention with dihydroartemisinin-piperaquine, a substantial proportion of children carried *Plasmodium* parasites at the end of the 30-day intervention period. When assessed by rapid dipstick tests, this proportion may be overestimated because the *P. falciparum* HRP2 protein can persist in circulation for several weeks after parasite clearance [52, 53]. We nonetheless found a similar prevalence estimate by microscopy, suggesting recrudescence of infections or the occurrence of new infections. In 2015, WHO revised the recommended target oral doses for malaria treatment with dihydroartemisinin and piperaquine in children, in recognition that the previously recommended dosage schedule (which was used in the present study) may be inadequate and may predispose children to an increased risk of treatment failure [54]. In addition, poor adherence to the second and third doses of dihydroartemisinin-piperaquine administered by parents at home may also have contributed to the recurrence of malaria. Future studies should consider direct observation of adherence to all doses during the entire course.

We selected a relatively short 30-day intervention with iron in the expectation that pre-medication with dihydroartemisinin-piperaquine would prevent malaria during this period, with a long-term view that the protection afforded by repeated chemoprevention with this combination drug would allow time windows for safe administration of short courses of iron intervention. The duration of protection of a single course of dihydroartemisinin-piperaquine is likely to vary between individuals and populations, depending, among other things, on variance in absorption and disposition of piperaquine and levels of acquired immunity (and thus on age and frequency and duration of exposure to *Plasmodium* infection). In a recent study among pre-school children in Burkina Faso, two cycles of chemoprevention with dihydroartemisinin-piperaquine, administered at the same target dose as in our study, resulted in a protection against malaria that persisted at a high level for 3 to 4 weeks and decreased rapidly thereafter, highlighting the importance of strict timing to ensure that children receive treatment at monthly intervals [55].

Our data show that the fortification dose of 3 mg iron as NaFeEDTA cannot be increased without a substantial proportion of children in this age range exceeding the ADI for EDTA. It has been argued, however, that this ADI may have been set too low [56], and a recent trial in Moroccan children has shown that daily oral intake of EDTA can reduce blood lead concentrations [57], which is important in view of the enormous public health burden due to lead exposure in developing countries.

The question may be raised whether our intervention period of 30 days was too short to show an effect on haemoglobin concentrations. In an earlier placebo-controlled randomised trial among Kenyan children aged 2–36 months, it was shown with a smaller sample size (79 iron; 76 placebo) than the present study that weekly supplementation with 6 mg elemental iron as ferrous fumarate per kilogram body weight improved haemoglobin concentration at 4 weeks after the start of intervention [58]. Several other trials with longer intervention periods of home fortification also failed to demonstrate haematological response to home fortification with iron. For instance, a randomised trial in 6-month old Kenyan infants showed no effect of daily home fortification with 2.5 mg iron as EDTA on haemoglobin concentration after 6 and 12 months of intervention [59]. Similarly, a trial conducted among Ghanaian children aged 8–20 months failed to show an effect on haemoglobin concentration after 6 months of daily home fortification with 40 mg elemental iron as microencapsulated ferrous fumarate [60]. These findings suggest that there are other underlying factors that may cause a lack of effect of iron interventions on haemoglobin concentration.

Inflammation was highly prevalent and is known to reduce iron absorption. In our subgroup analysis, however, there was no evidence that inflammation at baseline influenced the magnitude of the effect of iron interventions on haemoglobin concentration, and only weak evidence (*p* = 0.17) that it decreased the effect of home fortification with 12.5 mg iron as ferrous fumarate on plasma ferritin concentration. However, the cut-off definition for inflammation levels has not been validated in children, so there is a possibility that iron absorption was impaired at levels of inflammatory markers within the normal range (plasma CRP concentration <5 mg/L or plasma *α*_1_-acid glycoprotein concentration <1.0 g/L). Chronic infections caused by either viruses or low bacterial and parasitic loads can increase the inflammatory cytokine-mediated production of hepcidin, thus blocking iron absorption, in the absence of evident inflammation (Rita Wegmuller, personal communication, 25 May 2016). The notion that infection-induced inflammation can reduce iron absorption is supported by the findings of a study conducted among Gambian children aged 18–36 months, which showed elevated serum CRP concentrations and impaired absorption of orally supplemented iron in children with post-malarial anaemia compared with those with non-malarial anaemia, but serum CRP concentrations had reversed and iron absorption was recovered at 2 weeks after antimalarial treatment [61]. Another study among Kenyan infants showed that both low doses (2.5 mg iron as NaFeEDTA) and a high dose (12.5 mg iron as ferrous fumarate) increased the pathogenic profile of gut bacteria and gut inflammation [7].

Other factors that may have contributed to our failure to show efficacy are high contents of iron-inhibiting factors such as phytates and polyphenolic compounds in the food vehicles used for home fortification and suboptimal adherence to the daily home fortification with iron-containing powders.

The use of placebo in non-inferiority trials is controversial. Some argue that it is unethical to use placebos in the context of established interventions that have been shown to be efficacious [62, 63]. The Declaration of Helsinki (2013) asserts that ‘the benefits, risks, burdens and effectiveness of a new intervention must be tested against those of the best proven intervention(s)’ [64]. In our case, there was an established WHO recommendation for daily home fortification with 12.5 mg iron as ferrous salt, based on a meta-analysis of six randomised controlled trials [3] that showed an overall reduction of the prevalence of anaemia.

We included a third arm with placebo, however, because we were concerned that established efficacious interventions do not consistently demonstrate superiority in placebo-controlled studies. Notably, one-third of meta-analyses that demonstrate a protective effect from interventions are not supported by subsequent large randomised controlled trials [65, 66]. In addition, in the meta-analysis that formed the basis for the WHO recommendation for daily home fortification, three of six trials included could not exclude the absence of an effect on haemoglobin concentration. A trial among Ghanaian children, published after the meta-analysis but before we started our study, with a larger sample size than all previous studies combined, also failed to showed an effect of daily home fortification with 12.5 mg iron as ferrous fumarate on either change in haemoglobin concentration or the prevalence of anaemia, despite 45% of children being iron deficient at baseline and good adherence to intervention [61]. Our use of a three-armed non-inferiority trial that includes a placebo arm is consistent with international recommendations [67] and is validated by our results: in the absence of a placebo group, we would erroneously have concluded that 3 mg iron as NaFeEDTA was non-inferior to 12.5 mg iron as encapsulated ferrous fumarate.

In our meta-analysis, we found a high level of heterogeneity in effects across trials. How should we interpret this finding? In the absence of evidence for an effect in single trials, meta-analysis is often understood to be the continuation of the pursuit of statistical significance by other means. A high level of heterogeneity indicates, however, that there is no single true effect in a single, common population that underlies the trials included in the meta-analysis. Heterogeneity may reflect methodological differences between trials (dosage, formulation and duration of intervention, adherence, study quality, etc.), but it may also indicate that there are different types of populations, each with different true underlying effects. Thus, the pooled random effect may not reflect the actual effect in any particular population being studied, and has little value other than perhaps providing some evidence to inform policy decisions. Our meta-analysis (Additional file 2: Figure S4) suggests a small gain in haemoglobin concentration in most trials, indicating that home fortification with iron-containing micronutrient powders provides some benefit across different settings. This gain may be insufficient to recommend home fortification in all settings, as illustrated by the main results of our trial. Our finding of heterogeneity between trial results should stimulate subgroup analysis or meta-regression to identify population-specific factors that determine efficacy (e.g. differences in prevalence of iron deficiency and inflammation, food content of compounds that inhibit iron absorption). Such approaches may become possible as evidence is accrued from a variety of studies in different settings.

## Conclusions

In this population, home fortification with either 3 mg iron as NaFeEDTA or 12.5 mg iron as encapsulated ferrous fumarate was insufficiently efficacious to assess non-inferiority of 3 mg iron as NaFeEDTA compared to 12.5 mg iron as encapsulated ferrous fumarate. Our finding of heterogeneity between trial results should stimulate subgroup analysis or meta-regression to identify population-specific factors that determine efficacy.

## Additional files

Additional file 1: Statistical analysis plan. (DOCX 109 kb)

Additional file 2: Effect of home fortification with iron-containing powders on anaemia and haemoglobin concentration in pre-school children: meta-analysis of randomised controlled trials. (DOCX 61 kb)

Additional file 3: Effect of daily home fortification with iron on haemoglobin concentration by intention-to-treat analysis. (DOCX 16 kb)

## Abbreviations

ADI: Acceptable daily intake
AGP: α_1_-acid glycoprotein
CRP: C-reactive protein
EDTA: ethylenediaminetetraacetate
EMA: European Medicine Agency
HF-TAG: Home Fortification Technical Advisory Group
HRP2: histidine-rich protein 2
MEMS: Medication Events Monitoring System
pLDH: *Plasmodium* lactate dehydrogenase
