# Supplementary material for: Daily home fortification with iron as ferrous fumarate versus NaFeEDTA: a randomised, placebo-controlled, non-inferiority trial in Kenyan children
Source: BMC Med. 2017 Apr 28;15:89. doi: 10.1186/s12916-017-0839-z (PMC5408380; doi:10.1186/s12916-017-0839-z)
Supplement: Supplementary file 1 — Statistical analysis plan. (DOCX 109 kb) [file 12916_2017_839_MOESM1_ESM.docx]

### Additional file 1: Statistical analysis plan

Emily Teshome, Hans Verhoef

Version 19^th^ May 2015

1. **Preamble**

This plan was developed to analyse data from the SEICK (Safe and Effective Iron for Children in Kenya) trial. The study received ethical clearance in Kenya (Kenyatta University National Hospital/University of Nairobi, #KNH-ERC/A/402)) and in England (London School of Hygiene and Tropical Medicine, #6503)), and was registered at ClinicalTrials.gov (NCT02073149). The principal features of the planned analysis were established as part of the trial proposal. The current plan provides an update, was finalised after data collection but before breaking the randomization code, and should take precedence over the trial proposal.

1. **Trial objectives**

The study was primarily conceived to assess non-inferiority of daily home fortification with 3mg iron as NaFeEDTA compared with 12.5 mg iron as encapsulated ferrous fumarate, with haemoglobin concentration at the end of the 30-day intervention period as the primary outcome.

The study proposal states the following objectives:

1. In children aged 12-36 months, to compare daily home fortification for 30 days with two iron formulations (3mg iron as NaFeEDTA versus 12.5 mg iron as encapsulated ferrous fumarate) regarding:
2. Haemoglobin concentration at the end of the 30-day fortification period ***(primary objective)***;
3. Iron status at the end of the fortification period;
4. Serum NTBI concentrations at 3 hours after ingesting the first fortificant dose;
5. Faecal calprotectin concentration at the end of the fortification period;
6. *P. falciparum* infection at the end of the fortification period as indicated by whole blood density of asexual parasites or the presence of antigenemia;
7. Adherence to intervention.
8. To compare daily home fortification for 30 days with iron (3mg iron as NaFeEDTA or 12.5 mg iron as encapsulated ferrous fumarate) versus placebo regarding:
   1. The indicators listed under objective 1a-f;
   2. Time to development of anaemia after cessation of iron fortification.
9. To evaluate the accuracy of self-reported adherence with home fortification, using medication events monitoring system as a gold standard.
10. To assess associations between major intestinal parasites and haemoglobin concentration at baseline.
11. To compare intervention effects of iron on intestinal biota.

Objectives 1c, 1d, 4 and 5 are conditional to sufficient resources still being found to conduct the laboratory analyses required, and will not be further discussed in this plan.

1. **Study type and key design features**

The study concerns an individually-randomised, controlled non-inferiority trial with three parallel arms, with participants randomised to daily home fortification for 30 days with sachets containing either a) 3 mg iron as NaFeEDTA (experimental treatment); b) 12.5 mg iron as encapsulated ferrous fumarate (positive control); or c) placebo (negative control).

Demonstration of non-inferiority in a trial with two arms can have two meanings only: both interventions are equally effective, or both interventions are equally ineffective against placebo. Thus, as per guidelines of the European Medicine Agency [1] and the International Conference on Harmonisation of Technical Requirements for Registration of Pharmaceuticals for Human Use [2], we included a third arm with placebo, which allows a) demonstration of superiority of home fortification with 3mg iron as NaFeEDTA over placebo (proof of efficacy); b) demonstration of superiority of the reference (12.5 mg iron as encapsulated ferrous fumarate) over placebo (proof of assay sensitivity); and c) demonstration that the home fortification with 3mg iron as NaFeEDTA retains most of the efficacy of the reference over placebo (proof of non-inferiority).

Haemoglobin concentration at baseline was strongly expected to be prognostic for haemoglobin concentration at the end of intervention. To achieve group balance in size and baseline haemoglobin concentration, randomisation was based on a stratified block design, with strata defined by baseline haemoglobin concentration class (<100 g/L and ≥100 g/L) and randomly sized permuted blocks of 6 or 9. Stratification at the stage of randomisation and adjustment for this stratification in the analysis was also expected to improve the efficiency of the analysis and the precision of the estimated intervention effect. Because of the short recruitment period, we did not expect blocks to be associated with haemoglobin concentration, even though we could not entirely exclude this possibility (e.g. due to seasonal changes in diet or malaria transmission). We did not conduct interim analyses and had no stopping guidelines.

**Figure S1:** **Summary of study design (not to scale)**.

1. **Sample size calculations**

The target sample size was 108 participants per group, which was determined using procedures and terminology for sample size calculations in non-inferiority trials as recommended by US Food and Drug Administration [3,4]

1. Based on a meta-analysis [5], we estimated the effect of 12.5 mg ferrous fumarate on haemoglobin concentration relative to placebo. The lower limit of the 95% CI thus obtained (9.3 g/L) was used as M_1_, the minimum anticipated effect of 12.5 mg ferrous fumarate in our trial (**Figure 2;** left panel).
2. Next, we set M_2_ as the margin specified to preserve 50% of the anticipated minimum effect of 12.5 mg ferrous fumarate anticipated reduction in the 3mg NafeEDTA test treatment. This margin (haemoglobin concentration of 4.7 g/L) can be interpreted as the largest loss of effect compared to 12.5 mg ferrous fumarate (inferiority) that would be acceptable, and also is below an effect for 5g/L haemoglobin concentration that we consider to be of minimum importance for public health.
3. We set the sample size so that the lower limit of the 95% CI around the difference in haemoglobin concentration between the two iron formulations (i.e. 12.5 mg ferrous fumarate and 3.0 mg iron as NaFeEDTA) would lie above M2 (Figure 1; right panel).

Peer reviewers or editors of scientific journals may demand that we report sample size calculations and statistical power. Although such calculations are critically important in planning a study, we consider statistical power to be an exclusively pre-trial concept, which is irrelevant and conceptually wrong in the interpretation of study results, based on assumptions that are not usually met during trial implementation, and prone to be misinterpreted by most readers [6,7]. Thus we intend to refrain from reporting sample size calculations, but we will report the method used to determine the non-inferiority margin.


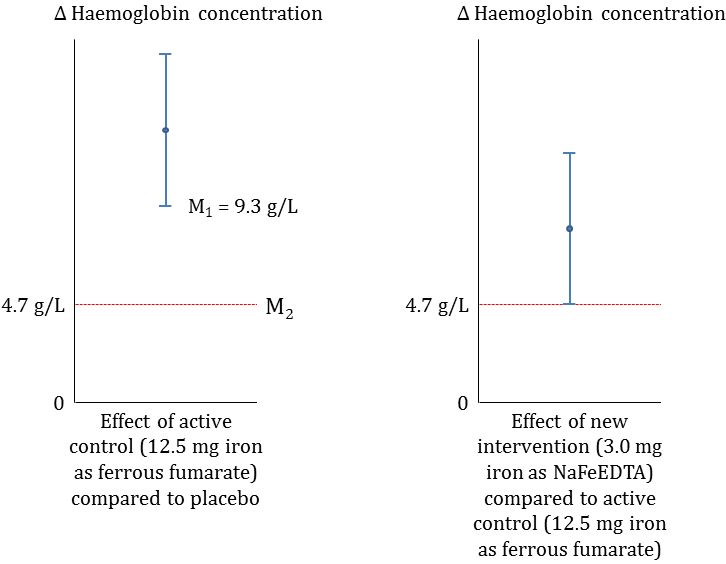


Figure S2: Theoretical framework for sample size determination

1. **Software**

Anthropometric indices will be calculated using WHO Anthro software vs.3.2.2 (World Health Organisation, Geneva, Switzerland). Data will be double entered, checked for completeness and verified for possible entry errors using Microsoft Excel. Data cleaning and analysis will be done using SPSS 21 (IBM, Armonk, NY); R-software version 3.2.0 ([www.r-project.org](http://www.r-project.org)) and CIA 2.2.0 (<http://www.som.soton.ac.uk/research/sites/cia/>and PowerView vs.3.5.2 (AARDEX Group ltd, Sion Switzerland) to analyse electronic adherence data.

1. **Intention-to-treat analysis versus per protocol analysis**

*Intention-to-treat (ITT) analysis:* Per definition, ITT analysis includes all randomised children. It generally is the preferred strategy for data analysis in superiority trials (objective 2 above) because a) it tends to avoid over-optimistic estimates of effect in real-life conditions, because non-compliers included in the ITT set will generally reduce the estimated treatment effect;[8] b) ITT analysis with multiple imputation yields valid results when missingness of data is random. Most trials aim to estimate the effects of allocating an intervention in real-life conditions, not the effects in the subgroup of the participants who adhere to it.

In a non-inferiority trial, however, intention-to-treat analysis is generally not conservative because protocol violation and non-adherence to treatment may cause the results of the treatment groups to appear similar [9].

*Per protocol analysis:* Per protocol analysis is restricted to participants who have adhered closely to what is prescribed in the study protocol in the terms of eligibility, interventions, and outcome assessment. It does not show the practical value of the experimental intervention, but rather envisages determining the biological effect of the intervention that can be obtained under ideal circumstances, with participants who adhere to trial instructions. However, missing outcome data due to attrition (drop-out) during the study or exclusions from the analysis raise the possibility that effect estimates obtained by per protocol analysis is biased. Per protocol analysis may also raise concerns about data manipulation.

In our study, we will include children in per protocol analysis who received at least one dose of home fortification, with exclusion of those not meeting inclusion criteria, poorly adhered to intervention, crossed over between intervention groups or ingested iron-containing preparations acquired outside the trial, and excluding those with missing outcome data. We will define adherence as the number of days that home fortificants have been made available relative to the scheduled 30-day intervention period, with availability indicated by the electronic device for storage of home fortificants having been opened. Poor adherence will be defined as adherence < 80% (23 days). This threshold is somewhat arbitrary, but it has been used in most published studies on medication adherence [10,11] and we believe that adherence above this threshold to be associated with improved iron status. Group adherence will be reported as the proportion of participants for whom home fortificants were available ≥80% (≥24 days).

*Relative importance of intention-to-treat analysis and per protocol analysis:* We conceived the SEICK trial primarily to be an explanatory trial (i.e. to assess intervention effects under optimal conditions) rather than a pragmatic trial (i.e. to assess intervention effects under real-life conditions). As per recommendations [12] we will pursue the primary (non-inferiority) objective by comparing results obtained by both intention-to-treat analysis and per protocol analysis. Non-inferiority will be accepted only if efficacy (objective #2), assay sensitivity (objective #2) and non-inferiority (objective #1) have all been shown. In our trial report, we will include results of the per protocol analysis in the main text, and results of the intention-to-treat analysis as supplementary material.

1. **Handling of outliers and missing data**

Outliers will be identified by examining a) ranges of continuous variables; b) frequency tables of categorical variables; c) visual inspection of scatter plots of pairs of continuous variables. Outliers will be dropped only if it is obvious that they are due to incorrectly entered or measured data.

Reasons for missing outcome data will be given where possible, coded and reported quantitatively as either a) withdrawal of consent; b) adverse events or withdrawn for medical reasons (reasons specified); c) no show for follow-up visit(s); d) moved away from the study area; e) refused intervention; f) refused to give human tissue samples; g) excluded from analysis due to not meeting inclusion criteria; h) excluded from analysis due to poor adherence to intervention; i) excluded from analysis due to crossing over between intervention groups; j) excluded from analysis due to taking iron-containing preparations acquired outside the trial; k) other reasons (specified).

For ITT analysis, missing data will be replaced under a missing-at-random assumption by multiple imputation (SPSS, ‘automatic’ imputation method; 20 iterations), with log-transformed variables as necessary to normalize distributions. Imputations will be done separately for each of the three intervention groups, with datasets derived with these imputed values being merged to allow subsequent analysis. All variables used in regression models will be included in the imputation model; these will include both baseline factors and outcomes. A list of these variables will be reported as supplementary material.

1. **Participant flow**

Figure 3 shows the format for presenting the flow of children during the four main stages of the trial (enrolment, allocation, follow-up and analysis). For children who were excluded before randomisation, reasons will be given where possible, coded and reported quantitatively as either a) no show; b) refused to participate; c) no written informed consent obtained; d) unlikely to stick to protocol; e) ineligible (with reasons specified if possible); f) medical conditions (with reasons specified if possible); g) other reasons; h) not known.

**Figure S3: Flow of study participant (incomplete)**

1. **Description of group characteristics at baseline**

For continuous variables, we will visually inspect histograms to assess whether they are normally distributed within intervention groups, with log-transformation of variables with a lognormal distribution. Population characteristics (Table 1) will be reported by intervention group as proportions, means (SD), geometric mean (geometric SD), and median (25th- and 75th percentiles) as appropriate, with corresponding group size. We will not test for group differences for reasons given by Assmann et al. [13].

Table S1: Description of baseline characteristics

| Variable | Type | Units/categories |
| --- | --- | --- |
| Age | Continuous | months |
| Age class | Binary | 12-23 months, 24-36 months |
| Sex | Binary | %males : %females |
| Height-for-age z-score | Continuous | SD |
| Weight-for-height z-score | Continuous | SD |
| Weight-for-age z-score | Continuous | SD |
| Haemoglobin concentration | Continuous | g/L |
| Anaemia status | Ordinal | *Severe to moderate anaemia:* haemoglobin concentration <89g/L; *mild anaemia:* haemoglobin concentration 90-109g/L; *non-anaemic:* haemoglobin concentration ≥110g/L |
| Plasma ferritin concentration | Continuous | μg/L |
| Iron status | Nominal | *Deficient:* plasma ferritin concentration <12 µg/L regardless of presence or absence of infection or inflammation; *replete:* plasma ferritin concentration ≥12 µg/L in the absence of infection or inflammation; *uncertain:* plasma ferritin concentration ≥12 µg/L in the presence of infection or inflammation |
| Plasma soluble transferrin receptor concentration | Continuous | mg/L |
| Plasma C-reactive protein concentration | Continuous | mg/L |
| Plasma *α*_1_-acid glycoprotein concentration | Continuous | mg/L |
| Inflammation | Binary | Plasma concentrations of C -reactive protein (CRP) > 5mg/L and/or α_1_-acid glycoprotein (AGP) > 1g/L |
| Whole blood ZPP concentration | Continuous | μmol/mol haem |
| Erythrocyte ZPP concentration | Continuous | μmol/mol haem |
| *Plasmodium* parasite density (asexual and sexual forms) | Continuous | µL^-1^ |
| *Plasmodium* infection, by rapid dipstick tests | Nominal | No infection; *P. falciparum* (by HRP2 or *P. falciparum*-specific pLDH), other human *Plasmodium* spp. (by pLDH), mixed infection |
| Urinary hepcidin concentration * | Continuous | nmol/mmol creatinine |
| Plasma folate concentration * | Continuous | nmol/L |
| Plasma vitamin B_12_ concentration | Continuous | pmol/L |

* Funds permitting

1. **General issues in the analysis of intervention effects**

Effects are measured by comparing outcomes measured after a particular intervention with those measured if an alternative intervention has been given (counterfactual argument), thus focusing on comparison of end points (instead of before-after measurements within groups). We will estimate effects when possible; P-values, where reported, will be 2-sided. We will avoid hypothesis testing and interpretation of results as ‘significant’ or p<0.05 for reasons reported elsewhere [14,15].

We will describe outcome variables by intervention groups as proportions, means (SD), geometric means (geometric SD), and median (25^th^- and 75^th^ percentiles) as appropriate, with corresponding group sizes. For continuous outcomes, these estimates will be obtained by ANOVA.

Intervention effects will be assessed as absolute differences (proportions and means) or relative differences in geometric means (variables with lognormal distributions) between groups, with accounting for the stratified block design. To facilitate interpretation, odds ratios resulting from these analyses will be converted to differences in proportions. To allow extrapolation of results to other settings, we will also consider reporting relative effects for binary outcomes (i.e. proportion ratios). Outcomes that are not normally distributed, even after log-transformation, will be compared using non-parametric tests (e.g. Mann-Whitney U test, or Wilcoxon rank sum statistics)

1. **Analysis of the primary outcome (objective 1a and 2)**

*Primary analysis:* We will estimate the difference in haemoglobin concentrations at the end of the 30-day fortification period between groups of children allocated to different iron formulations. Non-inferiority will be rejected if the difference between groups is less than the non-inferiority margin.

*Secondary analysis* of the primary outcome will be as follows:

1. *Adjustment* *for selected baseline variables:* Biased effect estimates would occur if there are group imbalances in baseline factors that are prognostic for outcome. For baseline factors that are known to be strongly, or at least moderately, associated with the primary outcome, or for which there is a strong biological rationale for such an association, i.e. iron status, plasma soluble transferrin receptor concentration, and age, we will evaluate the role of such bias using stratified analysis and directly by multiple linear regression models. We also expect haemoglobin concentration at baseline to be prognostic for outcome but, because this will already be accounted for by adjusting for study design. In stratified analysis, we will estimate intervention effects in subgroups formed by these baseline factors (for iron status: see definition in Table 1; plasma soluble transferrin receptor concentration dichotomised with the median as cut-off value; and age in classes of children aged 12-23 months and 24-30 months) to allow an assessment of the validity of model assumptions. In the regression models, we will compare effect estimates with and without adjustment by baseline factors (plasma concentrations of ferritin and soluble transferrin receptor and age; as continuous variables). In the interpretation of data, we will give priority to the unadjusted effect, unless the adjusted effect is markedly different (>15%), in which case the adjusted effect will be considered the primary analysis.

*Subgroup analysis:* Because iron absorption is known to depend on iron status, we will consider proxy markers for iron status (haemoglobin concentration, plasma concentrations of ferritin and soluble transferrin) at baseline as potential modifiers for intervention effects.

Stratified analysis will be used to measure effect sizes within subgroups. Evidence for group differences in intervention effects will be formally investigated in a multiple regression model with main effects (for baseline factors and the intervention) and multiplicative interaction terms.

To define subgroups, baseline factors will be categorized (haemoglobin concentration class <100 g/L and ≥100 g/L; iron status [see classification above] and plasma soluble transferrin receptor concentration [dichotomised, with the median as threshold]). For iron status, we will restrict the analysis to a comparison of iron-deficient and iron-replete subgroups.

Categorisation of baseline factors has the advantage that results can be conveniently presented in a forest plot. It has the disadvantage, however, that the precision of the interaction depends on the number and position of the cut-off values used to categorise baseline factors [16]. We will also explore interactions with baseline factors as continuous covariates, using fractional polynomials [16]. Such a strategy is more powerful, especially at towards extreme ends of the covariate distribution where limited numbers of observations generally preclude detection of interaction, and it may reveal more about the nature of possible interactions. We will inspect scattergrams and conduct sensitivity analysis as needed to examine the influence of possible outliers on results.

Results of subgroup analyses will be interpreted cautiously for reasons reported elsewhere [13].

1. *Intention-to-treat analysis:* For binary outcomes, pooled estimates from multiple iterations may result in non-integer values. This precludes computation of confidence intervals for differences in proportions, and may be counterintuitive to readers not well versed in multiple imputation techniques. To handle this problem, we will calculate differences in means under the assumption of binary outcome variables having a Bernoulli distribution. Pooled chi-squared statistics for cross-tabulated data and associated p-values will be calculated as described by Van Buuren (2012), thus the p-values obtained are only crude estimates of the true values [17].
2. *Paired comparisons of iron groups with placebo (objective 2):* This analysis will be done as for the comparison between iron groups (see above). Superiority will be established if no effect is excluded from the 95% CI.
3. **Analysis of secondary outcomes (objectives 1b and 2)**

To assess intervention effects, we will conduct all three possible paired comparisons between the intervention groups. We will use plasma ferritin concentration as an outcome indicator of iron status, and soluble transferrin receptor as an outcome indicator of the iron demand by the erythrocytes (both measured at the end of the 30-day intervention period). We will conduct analysis with and without adjustment for baseline factors, as well as intention-to-treat analysis (see methods above).

Plasma ferritin concentration has the limitation that it can be enhanced by inflammation independently of iron status. Because our interest in plasma ferritin concentration is limited to its value as a marker of iron status, we will adjust all analysis for inflammation markers (plasma concentrations of C -reactive protein and α_1_-acid glycoprotein; both as continuous variables) measured at the end of the 30-day intervention period. Thus we will effectively measure effects on plasma ferritin concentration assuming no inflammation.

Similarly, plasma soluble transferrin receptor concentration can be elevated by an increased erythropoiesis under influence of malaria-induced haemolysis. Because our interest in plasma soluble transferrin receptor concentration is limited to its value as a marker of tissue iron demand, we will adjust all analysis for inflammation markers (plasma concentrations of C -reactive protein and α_1_-acid glycoprotein; both as continuous variables) measured at the end of the 30-day intervention period.

Stratified analysis will be used to measure effect sizes within subgroups. To assess effect modification directly, we will compare multiple logistic regression models with and without product terms, with categorical baseline factors entered as dummy-coded variables.

1. **Multiplicity**

Multiple comparisons to evaluate non-inferiority (section 6) or to measure effects on multiple secondary outcomes may raise concerns about multiplicity (i.e. occurrence of false-positive results). We consider statistical solutions (e.g. Bonferroni correction) to be inappropriate in our trial because a) the concept of multiplicity is rooted in hypothesis testing, which is not our framework for making inferences; b) we will not formally adjust for multiplicity because any absence or present effect differences will be interpreted by the confidence interval in the context of the set thresholds as defined in section 6 [14,15,12]; c) for an intervention that leads to multiple changes in related outcomes, results from multiple comparisons are mutually reinforcing, not mutually debasing [18] .

In case of substantial discordance in results from analyses to evaluate non-inferiority, we will consider the results to be indeterminate.

**References**

1. *Pre-authorisation evaluation of medicines for human use; guidelines on the choice of the non-inferiority margin.* Document reference EMEA/CPMP/EWP/2158/99. London, UK: European Medicines Agency; 2005.
2. *ICH Harmonised tripartite guideline: choice of control group and related issues in clinical trials E10.* International Conference on Harmonisation of Technical Requirements for Registration of Pharmaceuticals for Human Use (ICH); 2000. [<http://www.ich.org/fileadmin/Public_Web_Site/ICH_Products/Guidelines/Efficacy/E10/Step4/E10_Guideline.pdf>] Accessed 13 March 2017.
3. *Guidance for industry: non-inferiority trials.* US Department of Health and Medical Services/Food and Drug Administration; 2010. Available at: [<http://www.fda.gov/downloads/Drugs/Guidances/UCM202140.pdf>] Accessed 13 March 2017.
4. Schumi J, Wittes JT: **Through the looking glass: understanding non-inferiority.** *Trials* 2011, **12:**106.
5. Okebe JU, Yahav D, Shbita R, Paul M: **Oral iron supplements for children in malaria-endemic areas.** *Cochrane Database Syst Rev* 2011, **10:**CD006589.
6. Goodman SN, Berlin JA: **The use of predicted confidence intervals when planning experiments and the misuse of power when interpreting results.** *Ann Intern Med* 1994, **121:**200-206.
7. Senn S: **Power is indeed irrelevant in interpreting completed studies.** BMJ 2002;325:1304.
8. *ICH Harmonised tripartite guideline: statistical principles for clinical trials E9.* International Conference on Harmonisation of Technical Requirements for Registration of Pharmaceuticals for Human Use (ICH); 1998. Available at: [<http://www.ich.org/fileadmin/Public_Web_Site/ICH_Products/Guidelines/Efficacy/E9/Step4/E9_Guideline.pdf>] Accessed 13 March 2017.
9. Piaggio G, Elbourne DR, Altman DG, et al.: **Reporting of noninferiority and equivalence randomized trials: an extension of the CONSORT statement.** *JAMA* 2006, **295:**1152-60.
10. Ho PM, Bryson CL, Rumsfeld JS: **Medication adherence: its importance in cardiovascular outcomes.** *Circulation* 2009, **119:**3028-35.
11. Knafl G J, Schoenthaler A, Ogedegbe G: **Secondary analysis of electronically monitored medication adherence data for a cohort of hypertensive African-Americans.** *Patient Pref Adherence* 2012, **6:**207–219.
12. *Points to consider on multiplicity issues in clinical trials.* EMA, Committee for Proprietary Medicinal Products (CPMP). CPMP/EWP/908/99. London, UK: European Medicine Agency; 2002.
13. Assmann [SF,](javascript:void(0);) Pocock [SJ](javascript:void(0);) , Enos LE, Kasten LE: **Subgroup analysis and other (mis)uses of baseline data in clinical trials.** *Lancet* 2000, **355:**1064–69.
14. Altman DG, Bland JM: **Absence of evidence is no evidence of absence.** *BMJ* 1995, **311:**485.
15. Sterne JAC, Smith Davey G: **Sifting the evidence—what's wrong with significance tests?** *BMJ* 2001, **322:**226–31.
16. Royston P, Sauerbrei W: **A new approach to modelling interactions between treatment and continuous covariates in clinical trials by using fractional polynomials.** *Stat Med* 2004, **23:**2509-25.
17. Van Buuren S: *Flexible imputation of missing data.* Interdisciplinary Statistics Series (Keiding N, Morgan BJT, Wikle CK, Van der Heijden P, eds.). Boca Raton, FL: Chapman and Hall/CRC; 2012: 159.
18. Schulz KF, Grimes DA: **Multiplicity in randomised trials I: endpoints and treatments.** *Lancet* 2005; **365:**1591–95.
