# Supplementary material for: Daily home fortification with iron as ferrous fumarate versus NaFeEDTA: a randomised, placebo-controlled, non-inferiority trial in Kenyan children
Source: BMC Med. 2017 Apr 28;15:89. doi: 10.1186/s12916-017-0839-z (PMC5408380; doi:10.1186/s12916-017-0839-z)
Supplement: Supplementary file 2 — Effect of home fortification with iron-containing powders on anaemia and haemoglobin concentration in pre-school children: meta-analysis of randomised controlled trials. (DOCX 61 kb) [file 12916_2017_839_MOESM2_ESM.docx]

**Additional file 2: Effect of home fortification with iron-containing powders on anaemia and haemoglobin concentration in preschool children: meta-analysis of randomised controlled trials.**

**Objective:** We conducted a meta-analysis of randomised controlled trials in preschool children to assess the effect of home fortification with iron-containing powders on haemoglobin concentration at the end of intervention.

**Methods:** This study is an update of the meta-analysis by Salam et al. [1], which included trials that provided iron-containing micronutrient powders either in the home or at designated centres, with different dosages and duration of intervention. Studies that included supporting interventions such as nutrition education were included only if the supporting interventions were given to both the intervention

and comparison groups, so that the difference between the two groups was solely of micronutrient powders. We did not follow a review protocol.

To identify studies that were published after the review by Salam et al. [1], we conducted a search on Pubmed (<https://www.ncbi.nlm.nih.gov/pubmed>) with the following terms: (Micronutrient* OR ”multiple micronutrient*” OR “multi-vitamin*” OR “multi-mineral*” OR “micronutrient powder*” OR MNP OR sprinkle*) AND (Fortifi* OR “food fortifi*” OR “point of use” OR “home fortification”) AND (hemoglobin OR haemoglobin OR anemia OR anaemia) and with filters: Randomized Controlled Trial; Publication date from 2012/11/01 to 2017/03/14; Humans; Infant: 1-23 months; Preschool Child: 2-5 years. This procedure yielded 21 papers. Upon screening titles and abstracts, 15 papers were rejected because they did not meet the criteria for inclusion in the present review. We perused the full text of the remaining 6 papers, and restricted ourselves to trials that used randomisation at the individual or cluster level. We included studies that reported home fortification in children aged < 5 years for at least 5 days/week with powders containing ≥10mg iron as ferrous salt (i.e. 80% of the 12.5mg iron recommended by the WHO for home prevention), or ≥2.0 mg iron as NaFeEDTA (considering that fractional absorption of iron added to foods as NaFeEDTA may be 4-5-fold higher than when added as ferrous salts [2,3]). We excluded trials with fortificants that were lipid-based or in liquid formulations. Authors were contacted to request additional data as necessary.

For each study, we restricted the analyses to haemoglobin concentration measured at the end of intervention or change of haemoglobin concentration over time, without adjustment for group differences in baseline factors that were predictive for outcome, and without adjustments for multiplicity. Differences in means with corresponding standard errors were calculated as described by Higgins et al. [4]. Cut-off points to define anaemia were as reported by the investigators (haemoglobin concentration < 110 g/L). For cluster-randomised trials, standard errors were inflated by the square root of the estimated design effect, *D*. Such estimates were obtained from published reports; if not reported, we used the formula $D=1+(m-1)\cdot ICC$, where *m* and *ICC* are the reported average cluster size and the intra-cluster- correlation coefficient, respectively. The *ICC* was estimated at 0.13 [5]). The analysis was implemented using the ‘metafor’ package [6] in R software vs. 3.2.0 ([www.r-project.org](http://www.r-project.org)). We used random-effects models, with Hartung and Knapp adjustment to account for the relatively small number of trials included in the analysis [7], and a restricted maximum-likelihood estimator of the between-study variance, τ^2^. We assessed potential reporting bias by visual inspection for asymmetry of the funnel plot, and Egger’s regression test.

**Results:** The study selection process is summarised in **Figure 1**. From the meta-analysis by Salam et al. [1], we excluded two studies that were conducted in school children older than 5 years [8,9]. For the study by Giovanni et al. [10], we pooled we the two groups that received iron. In the study by Sharieff et al. [11], we included the group that received micronutrients with heat-inactivated *Lactobacillus acidophilus*, which we pooled with the group that received these micronutrients without *L. acidophilus*.

From our Pubmed search, we excluded three papers because the intervention concerned a specially prepared, pre-fortified complementary food [12], children received only 120 sachets with micronutrient powders for flexible consumption over a supplementation period of 11 months [13], or infants in control group received a liquid iron preparation [14]. Thus we identified and included 16 eligible trials (including our own) in our meta-analysis [10,11,15-27].

The effect on haemoglobin concentration was highly heterogeneous (*I*^2^: 84.1%; p-value for test of heterogeneity: < 0.0001; **Figure 2**). The pooled effect on haemoglobin concentration was 3.9 g/L (95% CI: 2.2-5.5 g/L), indicating that, in random sample of a hypothetically infinite number of trials, each estimating a different true underlying effect, one may on average expect an increase in haemoglobin concentration by 3.9 g/L, with the 95% CI excluding an effect beyond 5.5 g/L. Visual inspection of the funnel plot (**Figure 3**) and Egger’s regression test (p=0.44) did not yield evidence for reporting bias.

**Discussion:** We found a high level of heterogeneity in effects across trials. How = should we interpret this finding? In the absence of evidence for an effect in single trials, meta-analysis is often understood to be the continuation of the pursuit of statistical significance by other means. A high level of heterogeneity indicates, however, that there is no single true effect in a single, common population that underlies the trials included in the meta-analysis. Heterogeneity may reflect methodological differences between trials (dosage, formulation and duration of intervention, adherence, study quality, etc.) but it may also indicate that there are different types of populations, each with different true underlying effects. Thus the pooled random-effect may not reflect the actual effect in any particular population being studied, and has little value other than perhaps providing some evidence to inform policy decisions. Reassuringly, our meta-analysis (Figure 1) suggests a small gain in haemoglobin concentration in most trials, indicating that home fortification with iron-containing micronutrient powders provides some benefit across different settings. Our trial results illustrate, however, that the evidence may be insufficient to recommend home fortification in all settings. Our finding of heterogeneity should stimulate subgroup analysis or meta-regression to identify population-specific factors that determine efficacy (e.g. differences in prevalence of iron deficiency and inflammation, food content of compounds that inhibit iron absorption). Such approaches may become possible as evidence is accrued from a variety of studies in different settings.

**References**

Salam RA, MacPhail C, Das JK, Bhutta ZA. **Effectiveness of Micronutrient Powders (MNP) in women and children.** *BMC Public Health* 2013; **13 Suppl 3:**S22.

Troesch B, Egli I, Zeder C, Hurrell RF, de Pee S, Zimmermann MB: **Optimization of a phytase-containing micronutrient powder with low amounts of highly bioavailable iron for in-home fortiﬁcation of complementary foods.** *Am J Clin Nutr* 2009, **89:**539–544.

1. Hurrel RF: **Fortiﬁcation: overcoming technical and practical barriers, forging effective strategies to combat iron deﬁciency.** *J Nutr* 2002, **132:**806S–812S.
2. Higgins J, Green S: *Cochrane handbook for systematic reviews of interventions version 5.0.2.* Cochrane Collaboration; 2008. [updated September 2009]
3. Verhoef H: *Iron deficiency and malaria as determinants of anaemia in African children.* PhD thesis. Wageningen, The Netherlands: Wageningen University; 2001. Available from: <http://edepot.wur.nl/197084> (accessed 30 January 2017).
4. Viechtbauer W: **Conducting meta-Analyses in R with the metafor package.** *J Stat Soft* 2010, **36:**1-48.
5. Guolo A, Varin C: **Random-effects meta-analysis: the number of studies matters.** *Stat Methods Med Res* 2015 (Published online May 7 before print, doi: 10.1177/0962280215583568).

Kumar VM, Rajagopalan S. **Trial using multiple micronutrient food supplement and its effect on cognition.** *Indian J Pediatr* 2008, **75:**671-78.

1. Osei AK, Rosenberg IH, Houser RF, Bulusu S, Mathews M, Hamer DH. **Community-level micronutrient fortification of school lunch meals improved vitamin A, folate, and iron status of schoolchildren in Himalayan villages of India.** *J Nutr* 2010, **140:**1146-1154.
2. Giovannini M, Sala D, Usuelli M, Livio L, Francescato G, Braga M, Radaelli G, Riva E: **Double-blind, placebo-controlled trial comparing effects of supplementation with two different combinations of micronutrients delivered as sprinkles on growth, anaemia, and iron deficiency in Cambodian infants.** *J Pediatr Gastroenterol Nutr* 2006, **42:**306-312
3. Sharieff W, Bhutta Z, Schauer C, Tomlinson G, Zlotkin S: **Micronutrients (including zinc) reduce diarrhoea in children: The Pakistan Sprinkles Diarrhoea Study.** *Arch Dis Child* 2006a, **91:**573–579.
4. Glinz D, Hurrell RF, Ouattara M, Zimmermann MB, Brittenham GM, Adiossan LG, Righetti AA, Seifert B, Diakité VG, Utzinger J, N'Goran EK, Wegmüller R. **The effect of iron-fortified complementary food and intermittent preventive treatment of malaria on anaemia in 12- to 36-month-old children: a cluster-randomised controlled trial.** *Malar J* 2015, **14:**347.
5. Bilenko N, Fraser D, Vardy H, Belmaker I. **Impact of multiple micronutrient supplementation ("sprinkles") on iron deficiency anemia in Bedouin Arab and Jewish infants.** *Isr Med Assoc J* 2014, **16:**434-438.

Osei AK, Pandey P, Spiro D, Adhikari D, Haselow N, De Morais C, Davis D. **Adding multiple micronutrient powders to a homestead food production programme yields marginally significant benefit on anaemia reduction among young children in Nepal.** *Matern Child Nutr* 2015, **11 Suppl 4:**188-202.

1. Adu-Afarwuah S, Lartey A, Brown KH, Zlotkin S, Briend A, Dewey KG: **Randomized comparison of 3 types of micronutrient supplements for home fortification of complementary foods in Ghana: effects on growth and motor development.** *Am J Clin Nutr* 2007, **86:**412-420.
2. Barth-Jaeggi T, Moretti D, Kvalsvig J, Holding PA, Njenga J, Mwangi A, Chhagan MK, Lacroix C, Zimmermann MB: **In-home fortification with 2.5 mg iron as NaFeEDTA does not reduce anaemia but increases weight gain: a randomised controlled trial in Kenyan infants.** *Matern Child Nutr* 2015, **11 Suppl 4:**151-162.
3. Jack SJ, Ou K, Chea M: **Effect of micronutrient Sprinkles on reducing anemia: a cluster-randomized effectiveness trial.** *Arch Pediatr Adolesc Med* 2012, **166:**842-850.
4. Kounnavong S, Sunahara T, Mascie-Taylor CG, Hashizume M, Okumura J, Moji K, Boupha B, Yamamoto T: **Effect of daily versus weekly home fortification with multiple micronutrient powder on haemoglobin concentration of young children in a rural area, Lao People's Democratic Republic: a randomised trial.** *Nutr J* 2011, **10:**129.
5. Lundeen E, Schueth T, Toktobaev N, Zlotkin S, Hyder SM, Houser R: **Daily use of Sprinkles micronutrient powder for 2 months reduces anemia among children 6 to 36 months of age in the Kyrgyz Republic: a cluster-randomized trial.** *Food Nutr Bull* 2010, **31:**446-460.
6. Macharia-Mutie CW, Moretti D, Van den Briel N, Omusundi AM, Mwangi AM, Kok FJ, Zimmermann MB, Brouwer ID: **Maize porridge enriched with a micronutrient powder containing low-dose iron as NaFeEDTA but not amaranth grain flour reduces anemia and iron deficiency in Kenyan preschool children.** *J Nutr* 2012, **142:**1756-1763.
7. Menon P, Ruel MT, Loechl CU, Arimond M, Habicht JP, Pelto G, Michaud L: **Micronutrient Sprinkles reduce anemia among 9- to 24-mo-old children when delivered through an integrated health and nutrition program in rural Haiti.** *J Nutr* 2007, 137:1023-1030.
8. Sazawal S, Dhingra P, Dhingra U, Gupta S, Iyengar V, Menon VP, Sarkar A, Black RE. **Compliance with home-based fortification strategies for delivery of iron and zinc: its effect on haematological and growth markers among 6-24 months old children in north India.** *J Health Popul Nutr* 2014, **32:**217-26.
9. Sharieff W, Yin S, Wu M, Yang Q, Schauer C, Tomlinson G, Zlotkin S: **Short-term daily or weekly administration of micronutrient Sprinkles has high compliance and does not cause iron overload in Chinese schoolchildren: a cluster-randomised trial.** *Public Health Nutr* 2006b, **9:**336-344.
10. Soofi S, Cousens S, Iqbal SP, Akhund T, Khan J, Ahmed I, Zaidi AK, Bhutta ZA: **Effect of provision of daily zinc and iron with several micronutrients on growth and morbidity among young children in Pakistan: a cluster-randomised trial.** *Lancet* 2013, **382:**29-40.
11. Suchdev PS, Ruth LJ, Woodruff BA, Mbakaya C, Mandava U, Flores-Ayala R, Jefferds MED, Quick R: **Selling Sprinkles micronutrient powder reduces anemia, iron deficiency, and vitamin A deficiency in young children in Western Kenya: a cluster-randomized controlled trial.** *Am J Clin Nutr* 2012, **95:**1223-1230.
12. Vama JL, Das S, Sankar R, Mannar MG, Levinson FJ, Hamer DH: **Community-level micronutrient fortification of a food supplement in India: a controlled trial in preschool children aged 36-66 mo.** *Am J Clin Nutr* 2007, **85:**1127-1133.
13. Zlotkin S, Newton S, Aimone AM, Azindow I, Amenga-Etego S, Tchum K, Mahama E, Thorpe KE, Owusu-Agyei S: **Effect of iron fortification on malaria incidence in infants and young children in Ghana: a randomized trial.** *JAMA* 2013, **310:**938-947.

**Figure 1: Study selection process**

**Figure 2: Effect of home fortification with iron-containing powders on haemoglobin concentration at end of intervention: meta-analysis of randomised controlled trials in preschool children ^1^**

Kounnavong et al. (2011) A and B refer to daily or twice-weekly home fortification with 10 mg iron, respectively. Soofi et al. (2013) A and B refer to home fortification with micronutrient powders excluding and including zinc. Teshome et al. A and B refer to effects of 12.5 mg iron as ferrous fumarate and 3 mg iron as NaFeEDTA, respectively, as reported by the present study.

^1^ τ^2^ (estimated amount of total heterogeneity): 9.55 (95% CI: 4.57, 23.86); *I*^2^ (total heterogeneity / total variability): 84.1% (95% CI: 71.7%, 93.0%); *H*^2^ (total variability / sampling variability): 6.29 (95% CI: 3.53, 14.21). Test for heterogeneity: Q (df = 18) = 102.61, p < 0.0001.

**Figure 3: Effect of home fortification with iron-containing powders on haemoglobin concentration at end of intervention: funnel plot of randomised controlled trials in preschool children**

Egger’s regression test for funnel plot asymmetry: p = 0.44
