# Supplementary material for: Daily home fortification with iron as ferrous fumarate versus NaFeEDTA: a randomised, placebo-controlled, non-inferiority trial in Kenyan children
Source: BMC Med. 2017 Apr 28;15:89. doi: 10.1186/s12916-017-0839-z (PMC5408380; doi:10.1186/s12916-017-0839-z)
Supplement: Supplementary file 3 — Effect of daily home fortification with iron on haemoglobin concentration by intention-to-treat analysis. (DOCX 16 kb) [file 12916_2017_839_MOESM3_ESM.docx]

**Additional file 3: Effect of daily home fortification with iron on haemoglobin concentration by intention-to-treat analysis.**

**Table S2. Effect of daily home fortification with 3mg iron as NaFeEDTA and 12.5mg iron as encapsulated ferrous fumarate on continuous outcomes at 30 days after start of intervention, by intention-to-treat analysis**

| **Outcome/intervention group** | **n** | **Estimate ^a^** | **Effect (95% CI) relative to placebo ^b^** | **Effect (95% CI) relative to standard ^b^** |
| --- | --- | --- | --- | --- |
| **Haemoglobin concentration** |  |  |  |  |
| Placebo | 112 | 107.0 g/L (1.3 g/L) | Reference | Not applicable |
| Iron, 3 mg as NaFeEDTA | 112 | 110.0 g/L (1.2 g/L) | 3.0 g/L (-0.1 g/L to 6.2 g/L) ^c^ | 1.3 g/L (-1.8 g/L to 4.3 g/L) ^c^ |
| Iron, 12.5 mg as ferrous fumarate | 114 | 108.6 g/L (1.2 g/L) | 1.6 g/L (-1.4 g/L to 4.7 g/L) ^c^ | Reference |
| **Plasma ferritin concentration** |  |  |  |  |
| Placebo | 112 | 29.5 μg/L | Reference | Not applicable |
| Iron, 3 mg as NaFeEDTA | 112 | 33.1 μg/L | 16.2% (-14.3% to 57.7%) ^d^ | 2.5% (-22.4% to 35.4%) ^d^ |
| Iron, 12.5 mg as ferrous fumarate | 114 | 32.5 μg/L | 12.3% (-17.1% to 52.0%) ^d^ | Reference |
| **Plasma soluble transferrin receptor concentration** |  |  |  |  |
| Placebo | 112 | 2.25 mg/L | Reference | Not applicable |
| Iron, 3mg as NaFeEDTA | 112 | 2.14 mg/L | -4.3% (-13.5% to 5.9%) ^d^ | 3.6% (-5.5% to 13.6%) ^d^ |
| Iron, 12.5mg as ferrous fumarate | 114 | 2.09 mg/L | -7.3% (-16.2% to 2.6%) ^d^ | Reference |
| **Erythrocyte ZPP-haem ratio** |  |  |  |  |
| Placebo | 112 | 137 μmol/mol [2.17] | Reference | Not applicable |
| Iron, 3mg as NaFeEDTA | 112 | 127 μmol/mol [1.97] | -6.5% (-23.5% to 14.2%) ^d^ | -5.3% (-21.7% to 14.5%) ^d^ |
| Iron, 12.5mg as ferrous fumarate | 114 | 135 μmol/mol [2.00] | -0.7% (-18.6% to 21.0%) ^d^ | Reference |
| ^a^ Mean (SE) or geometric mean; ^b^ Effects were adjusted for study design (blocks nested within strata of haemoglobin concentration <100 g/L and ≥100 g/L). ^c^ Effects were calculated as absolute difference in means. ^d^ Exponentiation of group differences with log-transformed outcomes resulted in associations being expressed as relative differences. | | | | |
